# Social Contact Structures and Time Use Patterns in the Manicaland Province of Zimbabwe

**DOI:** 10.1371/journal.pone.0170459

**Published:** 2017-01-18

**Authors:** Alessia Melegaro, Emanuele Del Fava, Piero Poletti, Stefano Merler, Constance Nyamukapa, John Williams, Simon Gregson, Piero Manfredi

**Affiliations:** 1 Carlo F. Dondena Centre for Research on Social Dynamics and Public Policy, Bocconi University, Milano, Italy; 2 Department of Policy Analysis and Public Management, Bocconi University, Milano, Italy; 3 Center for Information Technology, Bruno Kessler Foundation, Trento, Italy; 4 Department of Infectious Disease Epidemiology, Imperial College London, London, United Kingdom; 5 Biomedical Research and Training Institute, Harare, Zimbabwe; 6 Department of Economics and Management, University of Pisa, Pisa, Italy; Hokkaido University Graduate School of Medicine, JAPAN

## Abstract

**Background:**

Patterns of person-to-person contacts relevant for infectious diseases transmission are still poorly quantified in Sub-Saharan Africa (SSA), where socio-demographic structures and behavioral attitudes are expected to be different from those of more developed countries.

**Methods and Findings:**

We conducted a diary-based survey on daily contacts and time-use of individuals of different ages in one rural and one peri-urban site of Manicaland, Zimbabwe. A total of 2,490 diaries were collected and used to derive age-structured contact matrices, to analyze time spent by individuals in different settings, and to identify the key determinants of individuals’ mixing patterns. Overall 10.8 contacts per person/day were reported, with a significant difference between the peri-urban and the rural site (11.6 versus 10.2). A strong age-assortativeness characterized contacts of school-aged children, whereas the high proportion of extended families and the young population age-structure led to a significant intergenerational mixing at older ages. Individuals spent on average 67% of daytime at home, 2% at work, and 9% at school. Active participation in school and work resulted the key drivers of the number of contacts and, similarly, household size, class size, and time spent at work influenced the number of home, school, and work contacts, respectively. We found that the heterogeneous nature of home contacts is critical for an epidemic transmission chain. In particular, our results suggest that, during the initial phase of an epidemic, about 50% of infections are expected to occur among individuals younger than 12 years and less than 20% among individuals older than 35 years.

**Conclusions:**

With the current work, we have gathered data and information on the ways through which individuals in SSA interact, and on the factors that mostly facilitate this interaction. Monitoring these processes is critical to realistically predict the effects of interventions on infectious diseases dynamics.

## Introduction

A vast epidemiological literature has shown the importance of considering heterogeneous social contact structures when investigating the transmission of airborne infections and the effects of possible control measures such as vaccination. As a consequence, mathematical modeling of infectious diseases, initially based on simplifying theory-driven assumptions [1] such as homogeneous mixing, have gradually shifted towards using empirical evidence on real individuals’ interactions. In particular, over the past decade, field data on social contacts patterns have been gathered through diary-based surveys for a number of countries in Europe [2–7], North America [8], Oceania [9], Asia [6,10–14], South America [15], and Africa [16–18]. Age-specific mixing matrices built on the gathered data have been largely used to model the spread of epidemics driven by close-contact interactions [19–22], and the transmission of endemic childhood infections [23–27]. In addition to these field studies, agent-based modeling techniques have recently been employed to derive synthetic contact matrices for European countries from detailed country-specific socio-demographic data [28,29].

However, two important challenges need to be further explored to better identify sources of heterogeneity in human mixing patterns. The first one regards the poor knowledge of social contact structures in regions, such as sub-Saharan Africa (SSA), characterized by a dramatically high burden of infectious diseases and an unstable and complex socio-demographic context [30]. A second challenge regards the understanding of what type of individual socio-demographic and behavioral features are associated with a given contact pattern. Past modeling efforts have suggested that age is sufficient to capture the main differences in individual social attitudes. However, while in developed countries age may appropriately reflect personal daily routines and activities, it is still unclear whether this is also valid for developing regions and, more generally, which are the key determinants for human interactions in different settings.

To address these questions, we conducted a field study in Zimbabwe, a SSA country characterized by a slowly progressing demographic transition [30], and gathered data on contact patterns and individuals daily routines in the Manicaland province, a predominantly rural setting, but with growing urban settlements. As part of the study, we measured the number and the location of contacts, along with the age of contactees and with socio-demographic characteristics of households, schools and work settings. In addition, and for the first time to our knowledge, information on daily routines, i.e., on time spent at home, school, work, and in the general community, were also gathered from the same individuals. Time-Use studies have extensively been used to address demographic research questions [31,32]. However, they have also shown to provide complementary information on contact data to understand the spread of infections [33,34] and to be very useful for parameterizing agent-based models for infection transmission [28,35].

The combination of contact and time use data differentiates this study from previous work and enables us to better understand the heterogeneity in mixing patterns in a typical low-income setting and to identify which components of the individual’s behavior affect the level of interactions with the rest of the community. A deeper investigation on the ways individuals mix, and on the possible socio-demographic drivers, can increase, even in these settings, the robustness of epidemiological models aimed at assessing effective and cost-effective intervention policies for infectious diseases control.

## Materials and Methods

### Study Population

The study was conducted as part of the sixth round of the Manicaland HIV/STD Prevention Study [36], a general population cohort study carried out between 1998 and 2013 in Manicaland, the easternmost province of Zimbabwe. Our target population consisted of the population of two sites among those already involved in the Manicaland HIV Study, selected as the most extreme ones in terms of urbanization: a small peri-urban township, situated within one of the main tourist areas in Zimbabwe, and a roadside trading center, characterized by a tarred road passing through the village and surrounded by smaller subsistence farming villages, all scattered around the center (population sizes were, respectively, 4,836 and 6,733, in 2012). Compared to the whole country, the rural site is representative of rural Zimbabwe in terms of age distribution and household size, whereas the peri-urban site can be considered as illustrative of a *rural—urban* transition zone. Further details on the study population can be found in S1 Text.

Individuals of all ages living in the two sites were considered eligible for inclusion in the study. The sample was stratified by age group (i.e., <1, 1–5, 6–12, 13–18, 19–34, 35–60, and >60) and site of residence. The age stratification was designed to mimic the local schooling system (pre-primary, primary, and secondary school), as well as to capture working-age adults and elderly people. Further details of the approach used for the selection of participants and their enrollment can be found in S1 Text. The study was approved by the Imperial College Research Ethics Committee, the Biomedical and Research Training Institute Institutional Review Board in Harare, and the Medical Research Council of Zimbabwe. All respondents older than 17 years old had to personally provide written informed consent to study participation. For younger participants, either one of the parent or the guardian had to provide written consent for participants. In addition, verbal assent was also required by participants aged between 13 and 17 years old.

### Contact and Time Use Survey

Respondents were asked to personally fill in a diary, prepared in English and translated into the local language (Shona), in order to report all the contacts that they had and where they spent their time during two consecutive, randomly assigned, days. Individuals’ age, education, occupational status along with details on participants’ household composition and on their schooling and working environments were also collected and complemented with records coming from the Manicaland HIV/STD Prevention Study [36]. In line with previous studies [4,10,11,15], a *contact* was defined as an interaction between two individuals, either physical (when involving skin-to-skin contact), or non-physical (when involving a two-way conversation with three or more words in the physical presence of another person, but no skin-to-skin contact). Respondents were requested to report both physical and non-physical contacts, separately. Multiple contacts with the same individual were reported only once per day. Moreover, for each encounter, respondents provided information on gender and age group of contactee (exact age when known), and on the social setting where the encounter occurred: i) the participant’s home, ii) school, iii) workplace, iv) general community (i.e., any remaining outdoor or indoor setting attended).

Respondents provided information on their use of time by recording all the visited settings during their day. Day time was divided into time slots (of 1 or 2 hours) reflecting the position of the sun during the day. For the early and late night time, longer time windows were considered (8pm–12pm; 12pm–4am).

Data were gathered from March 2013 to August 2013. During this period schools were closed for holidays from March 28th to May 6th. For illiterate adults and children aged less than 10 years, an additional individual, denoted hereafter as “shadow”, was chosen to fill in the questionnaire on behalf of the study participant. Results of a preliminary pilot study on a sample of 25 people, recruited in a different site from those used in the survey, were used to optimize the questionnaire. For this reason, these respondents were not included in the current sample.

### Data Analysis

Time use records are used to compute the proportion of individuals in the different settings in each time slot. The routine nature of daily activities is assessed by testing the correlation between the two consecutive days, using the Cramer’s V statistic for binary variables, considering individuals’ presence over time across the different settings.

Similarly to past work [3,24,37], an age-specific contact matrix (by five-years age bands), providing the average number of contacts reported by respondents in age group *i* with contactees in age group *j*, is computed. Multiple imputation techniques are used when the exact age of the contactee is not available, and bivariate smoothing is performed (see S2 Text). Age-assortativeness of the obtained contact matrices (i.e., preferred mixing among people of the same age) is assessed using the *Q* index [28], a measure representing departures from proportionate mixing, ranging from zero (proportionate) to one (fully assortative).

Generalized estimating equations (GEEs) are used to statistically identify the key determinants of the overall number of contacts of study participants, as well as of the individual number of contacts at home, at school, at work, and in the general community [38]. Possible explanatory variables include the time spent by individuals in the different settings, the socio-demographic characteristics of respondents (gender, age group, and site of residence), setting-related characteristics (individuals’ household, school, class, and workplace sizes), and the type of day (normal weekday or with school holiday, weekend) in which the diary was kept. Moreover, we test the impact of socio-economic conditions of households, which represents a different stratification of the population from the urbanization level of the site, based on a socio-economic status (SES) index constructed using house characteristics and owned assets [39]. Finally, we also test for the possible effect of seasonal changes in climatic conditions, using monthly data on rainfall and temperature. Statistical analyses are performed with Stata (version 14.0), and R (version 3.2.3). More details can be found in S3 Text.

### Epidemiological Implications of Social Contact Patterns

The epidemiological consequences of the estimated mixing patterns are evaluated by simulating the age distribution of individuals infected during the initial phase of an epidemic in a fully susceptible population. In particular, such distribution was obtained by computing the eigenvector of the next generation matrix associated with contacts, considered for any possible age of the index case in the community, and by assuming an age-independent transmission rate per contact, under the so-called “social contact hypothesis” [3]. It is worth noting that the age distribution of cases during the early phase of the epidemic depends neither on the choice of the duration of the infectivity period, nor on the considered value of the basic reproductive number R_0_. This means that, although the attack rate, the timing, and the severity associated with an infection are generally disease-specific, the impact of mixing patterns on the age distribution of cases within a susceptible population are mainly driven by the type of contacts relevant for infection transmission and the socio-demographic structure of the considered population.

## Results

### Sample Description

A total of 1,245 complete diaries were collected during the study period, 554 from the peri-urban site and 691 from the rural site, for a total of 2,490 person-days (Table 1). Of these daily diaries, 52% were completed during weekdays, 20% during weekends, and 28% during school holidays. Although the samples from the two sites appear similar in terms of age distribution, the peri-urban township is characterized by smaller households (HHs) (median HH size is 4 in the peri-urban site versus 5 in the rural one, *p* = 0.03), and a higher proportion of nuclear families (55% versus 43%, *p*<0.001). Moreover, significantly different levels of schooling and working are detected, with 34% and 19% of individuals reporting to be either students or workers in the peri-urban township, as opposed to 42% and 10% in the subsistence farming area, respectively.

**Table 1:** Characteristics of diaries, social contacts and time use. Number (and percentage) of daily diaries per site and overall, total number of contacts and median number of contacts (with IQR), and percentage of time spent in each setting, for socio-demographic characteristics of participants per day, Manicaland (Zimbabwe), 2013.

Variables	No. daily diaries (%)			Social contacts		Time Use			
	Peri-urban township	Subsistence farming area	Overall	No. total contacts	Median no contacts (IQR)	Home (%)	School (%)	Work (%)	General Community (%)
**Overall**	1108 (44.5%)	1382 (55.5%)	2490	26981	9 (6–14)	66.87	9.00	2.21	21.92
**Age group**									
0–5 yrs.	358 (32.3%)	394 (28.5%)	752 (30.2%)	6543	8 (5–12)	76.85	2.97	0.00	20.18
6–18 yrs. active	73 (6.6%)	281 (20.3%)	354 (14.2%)	5217	12 (8–16)	42.94	49.95	0.00	7.11
6–18 yrs. non-active	258 (23.3%)	251 (18.2%)	509 (20.4%)	5538	9 (6–14)	71.41	0.00	0.20	28.34
19–59 yrs. active	71 (6.4%)	23 (1.7%)	94 (3.8%)	1152	11 (7–14)	46.74	0.32	46.17	6.78
19–59 yrs. non-active	285 (25.7%)	368 (26.6%)	653 (26.2%)	7175	9 (5–14)	71.63	1.88	0.00	26.49
60+ yrs.	54 (4.9%)	62 (4.5%)	116 (4.7%)	1238	9 (5–14)	72.23	0.00	0.48	27.29
**Sex**									
Female	394 (35.6%)	560 (40.5%)	954 (38.3%)	9952	9 (6–14)	63.23	10.97	2.73	23.06
Male	608 (54.9%)	682 (49.3%)	1290 (51.8%)	14490	9 (6–14)	69.23	8.01	1.90	20.85
**Day type**									
Working days	340 (30.7%)	961 (69.7%)	1301 (52.3%)	14336	9 (6–14)	63.38	15.34	1.78	19.51
Weekends	274 (24.7%)	412 (29.9%)	686 (20.1%)	7062	9 (6–14)	71.55	1.83	1.35	25.27
School holidays	494 (44.6%)	5 (0.4%)	499 (27.6%)	5531	9 (6–14)	70.04	1.14	4.77	24.05
**HH size**									
1–3 pp.	290 (26.2%)	332 (24%)	622 (25.0%)	6573	8 (5–14)	63.97	9.03	3.25	23.75
4 pp.	280 (25.3%)	314 (22.7%)	594 (23.9%)	6057	9 (6–14)	65.11	9.43	1.88	23.59
5 pp.	224 (20.2%)	334 (24.2%)	558 (22.4%)	5813	9 (6–13)	69.56	8.40	2.60	19.45
6+ pp.	310 (28.1%)	400 (28.9%)	710 (28.5%)	8437	10 (7–14)	68.91	9.19	1.16	20.73
**SES index**									
Low	264 (23.8%)	528 (38.2%)	792 (31.8%)	8931	9 (6–14)	68.09	9.44	0.61	21.86
Medium	242 (21.8%)	580 (42%)	822 (33.0%)	8865	9 (6–14)	63.64	10.70	1.48	24.17
High	602 (54.3%)	274 (19.8%)	876 (35.2%)	9185	9 (6–14)	68.97	6.92	4.34	19.77
**Site**									
Peri-urban				12491	10 (6–14)	68.57	4.62	4.33	22.48
Rural				14490	9 (6–14)	65.65	12.12	0.70	21.53

### Time Use Patterns

**Fig 1:**
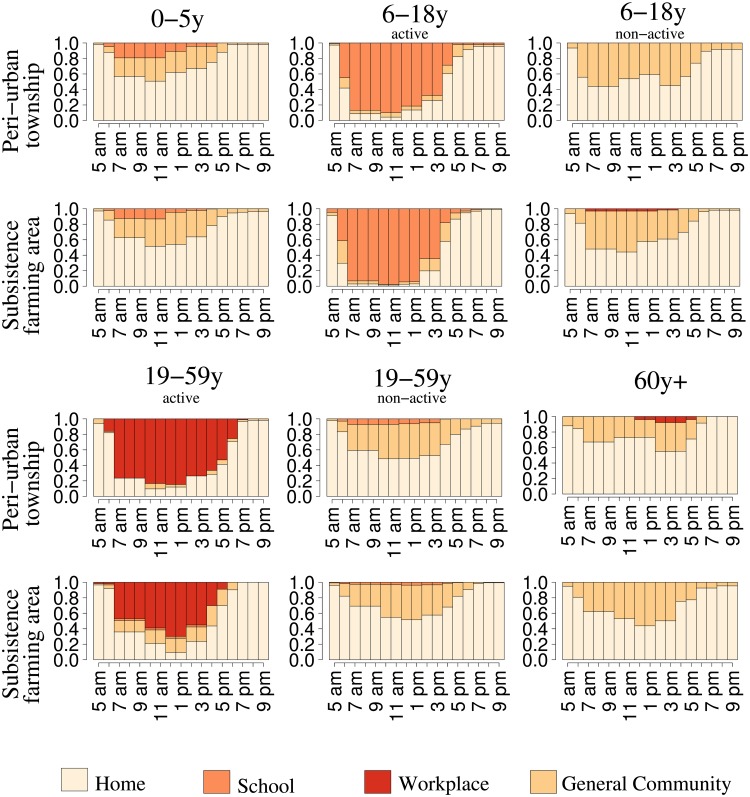
Patterns of time use by setting, age group, and site, Manicaland (Zimbabwe), 2013. Proportion of individuals present in different settings (home, school, workplace, general community) at different hours of the day (from 5 am to 10 pm) during a weekday (from Monday to Friday), stratified by age group and site of residence. The age groups 6–18 years and 19–59 years are stratified between *active* and *non-active* students and workers, respectively.

Individuals spent overall 66.9%, of their day time (i.e., 5am-10pm) at home, 9% at school, 2.2% at work, and 21.9% in the general community. The main differences in daily routines emerge when considering *active* versus *non-active* individuals, with *active* defined as those attending school or work for at least a single time slot (Fig 1). For the active people, the proportion of daily time spent at home and in the general community is much lower than for those non-active (Table 1). The recorded percentage of active students among school-age children is significantly higher in the rural site (53.5% versus 21.8%, p<0.001), reflecting both the different proportions of enrollment between sites (91.1% versus 84.4%, p<0.001) and the occurrence of school holidays in the peri-urban township. Despite this difference in actual school attendance, active school-age individuals in the two sites spend a similar amount of time at school (51% in the rural site versus 46.2% in the peri-urban site, p = 0.64). This does not hold for the time devoted to work, since in the peri-urban site we find a larger proportion of active workers in the age group 19–59 (21.5% versus 5.7% in the rural site, p<0.001), who also spend more of their daytime in the workplace (51.5% versus 30.9% in the rural site, p<0.001). A high correlation between time use patterns over the two days is found across all respondents (0.86, interquartile range: 0.77–0.96). Time use patterns on school holidays and weekends are shown in S4 Text.

### Number of Contacts by Age and Setting

**Fig 2:**
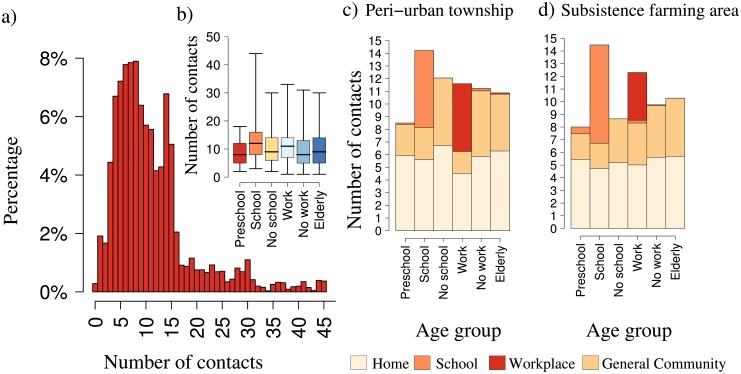
Number of contacts per participant, stratified by age and by activity class, Manicaland (Zimbabwe), 2013. a) Distribution of the overall number of contacts reported by participants. b) Boxplot (2.5%, 25%, 50%, 75%, and 97.5% quantiles) of the number of contacts, stratified by age group of participant and by active participation to school or work: preschool (0–5 yrs.), school and no school (6–18 yrs.), work and no work (19–59 yrs.), and the elderly (60+ yrs.). c) Average number of contacts per setting, stratified by age group of participant and active participation to school and work, in the peri-urban township. d) As c), but for the subsistence farming area.

A total of 26,981 different contacts was reported over the two survey days, resulting in an estimated average number of contacts of 11.1 (median 9, IQR 6–14) per person per day (most of which are *physical contacts*, S3 Text). The distribution of the number of contacts is highly right-skewed (Fig 2a), with 28% of people reporting 13 or more different contacts and accounting for 53% of total contacts.

The average number of contacts in the peri-urban site is 11.6 (median 10, IQR 6–14) versus 10.8 in the rural site (median 9, IQR 6–14) (*p*<0.001, median test). Interestingly, differences between sites are also significant among working-age adults and non-active people, and among individuals living in extended families (see Table A in S3 Text). Although some variations across age groups are present, with infants reporting a significantly lower average number of contacts (8.7, *p*<0.005), being active at school and, to a lesser extent, at work, are found to be the main determinants of the reported number of contacts (Fig 2). In particular, active students and workers report on average, respectively, 35% (*p*<0.001) and 12% (*p* = 0.10) more contacts than non-active individuals in the same age bands, but with a reduction of 17% (*p* = 0.001) of their home contacts.

### Age-Specific Mixing Matrices

**Fig 3:**
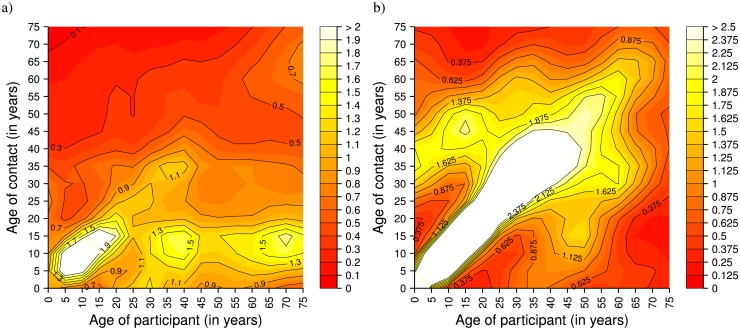
Comparison between social contacts in Manicaland (Zimbabwe) and in Italy. a) Social contact matrix derived for Manicaland (Zimbabwe), 2013, considering all reported contacts and applying bivariate smoothing. The matrix shows the average number of contacts during working days (weekends and school holidays are excluded) of participants in the *i-*th age group with individuals in the *j-*th age group. b) As a), but for Italy (data from the POLYMOD study [4], weekends excluded).

The derived social contact matrix for all reported contacts is characterized by a remarkable age assortativeness among children aged 5–19 years (Fig 3a), decreasing afterwards, except for a moderate rise among people aged 35–39 years. The low assortativeness characterizing adults with respect to what observed in European countries (e.g., Italy, Fig 3b) is also reflected by the relatively smaller value of the *Q* index associated with the matrix (0.051 in Manicaland versus 0.11 in Italy), computed excluding weekends and school holidays. Beyond the age-assortativeness mostly driven by school contacts, but also detectable in the general community (Fig C in S3 Text), the mixing structure is strongly affected by the young age distribution of the study population and by the presence of extended families, where mixing is predominantly homogeneous. Some evidence of intergenerational contacts can be noticed, and this is likely due to family contacts between parents/grandparents with children/grandchildren. Interestingly, matrix patterns characterizing individuals older than 20 years mainly reflect the structure of contacts observed at home.

These general patterns are confirmed when stratifying contacts by site (Fig D in S3 Text). However, the peri-urban township is characterized by a higher average number of work-related contacts among adults (30–49 years), a relatively higher intensity of contacts between parents and children, and a more assortative mixing in primary schools with little interactions across different ages, possibly indicating more homogeneous classes.

Additional differences characterizing mixing patterns based on alternative contact stratifications are shown in S3 Text.

### Determinants for the Number of Contacts

**Fig 4:**
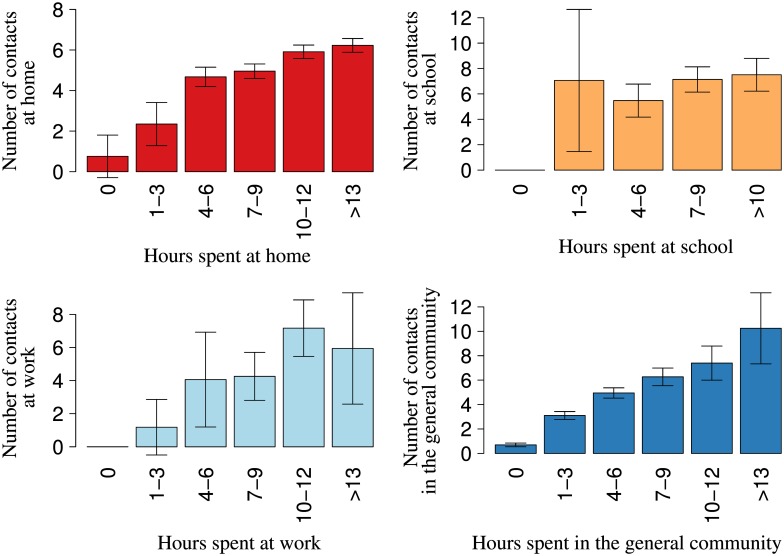
Number of social contacts per setting by total time spent. Average number of setting-specific contacts by total time spent (in hours) by participants in the respective setting: a) at home, b) at school, c) at work, and d) in the general community, Manicaland (Zimbabwe), 2013. For each group of time spent in the setting, we reported the 95% CI around the average.

The overall number of reported contacts is positively associated with the time individuals spend outside of their home (Table 2). In particular, the more time individuals spend at work and in the general community, the higher the number of contacts in these settings, and the lower the number of home contacts (Fig 4). The number of school contacts increases with the class size, but is not associated with the time spent at school. A significantly higher number of contacts in the general community are found in the peri-urban township as opposed to the rural area, probably the consequence of a wider social network and a higher proportion of working age adults. Better off individuals with medium and high SES, independently from the site where they reside, reported less social contacts at home than those with low SES. On the other hand, people living in larger households were associated with a higher number of home contacts. Infants and pre-school children reported a significantly lower number of overall, home, and general community contacts as opposed to school-aged individuals (6–18 years old). Conversely, working-age adults reported a significantly higher number of contacts in the general community than school-age children. Finally, we did not find any difference between different types of day (normal weekdays, weekdays with school holidays, and weekends), but we found an effect of the seasonal change in the climatic conditions, in particular, school contacts decrease in months characterized by higher rainfall. Since the class size is a major determinant of school contacts, this association might suggest that, in days of heavy rain, a lower number of children attend school, affecting the reported number of contacts in this setting. On the other hand, using a different climatic factor, i.e., the average monthly temperature, we did not find any effect (see Table B in S3 Text).”

**Table 2:** GEE model for the number of social contacts. Coefficients (with respective semi-robust standard error and significance at 5%) for the association between the number of social contacts (overall and by setting of contact), according to GEEs with negative binomial distribution, Manicaland (Zimbabwe), 2013.

Variable	Category	Overall	Home	School^a^	Work^a^^,^^b^	General Community
**Intercept**		2.03 (0.080)*	1.63 (0.089)*	0.75 (0.59)	1.18 (0.71)	1.08 (0.14)*
**Total time use (hours)**	*School*	0.052 (0.0060)*	-0.035 (0.0067)*	0.026 (0.025)	-	-0.0076 (0.015)
*Ref*.: *Home*	*Workplace*	0.040 (0.0098)*	-0.031 (0.012)*	-	0.14 (0.038)*	-0.026 (0.025)
	*General community*	0.051 (0.0047)*	-0.023 (0.0048)*	-0.041 (0.029)	0.095 (0.068)	0.090 (0.0089)*
**Site of residence**	*Peri-urban*	0.099 (0.053)	0.11 (0.059)	-0.088 (0.15)	-0.58 (0.47)	0.27 (0.091)*
**Gender**	*Female*	-0.13 (0.036)*	-0.18 (0.041)*	-0.012 (0.11)	-0.35 (0.28)	-0.050 (0.069)
**Age group**	*0–5 yrs*.	-0.23 (0.045)*	-0.14 (0.048)*	-0.22 (0.21)	-	-0.31 (0.087)*
*Ref*.: *6–18 yrs*.	*19–59 yrs*.	-0.026 (0.048)	-0.050 (0.052)	-0.57 (0.37)	-	0.17 (0.081)*
	*60+ yrs*.	-0.027 (0.10)	0.013 (0.13)	n.a.	-	0.19 (0.18)
**Household size**		0.035 (0.0093)*	0.079 (0.010)*	0.019 (0.035)	-0.19 (0.063)*	-0.022 (0.016)
**Socio-economic status**^c^	*Medium*	-0.088 (0.048)	-0.13 (0.054)*	-0.34 (0.14)*	0.68 (0.45)	-0.057 (0.080)
*Ref*.: *Low*	*High*	-0.083 (0.054)	-0.13 (0.063)*	-0.13 (0.14)	0.46 (0.32)	-0.12 (0.099)
**Class size**	*Size*	**-**	-	0.050 (0.020)*	-	**-**
	*Size squared*	**-**	-	-0.00049 (0.00024)*	-	**-**
**Workplace size**	*Size*	-	-	-	0.0095 (0.013)	-
	*Size squared*	-	-	-	-0.000061 (0.00008)	-
**Day type**	*School holiday*	0.0095 (0.051)	-0.068 (0.057)	0.049 (0.23)	0.29 (0.30)	0.13 (0.098)
*Ref*.: *Weekday*	*Weekend*	-0.0051 (0.039)	0.017 (0.041)	-0.15 (0.20)	0.36 (0.25)	0.051 (0.071)
**Average monthly rainfall**		0.0012 (0.0013)	0.0013 (0.0016)	-0.0077 (0.0026)*	-0.0031 (0.012)	-0.0013 (0.0023)
**Sample size**		2218	2207	380	62	1493

*: *p*<0.05.

^a^ For school and workplace, the analysis was performed on the subsamples of individuals who reported time use data for such settings.

^b^ *Total time use (school)*, and *Age* have been excluded from the model for the sake of convergence.

^c^ The *Socio-economic status* has been computed using data on house characteristics and owned assets.

A high positive correlation between individuals’ number of contacts over the two days was found (0.63). However, when stratifying by setting, we found large differences, as values ranged from almost zero for work contacts to 0.61 for home contacts. This shows the routine nature of home contacts as opposed to the randomness in work contacts, mostly in case of more informal occupations.

### Characteristics of an Epidemic Driven by the Estimated Contact Matrix

**Fig 5:**
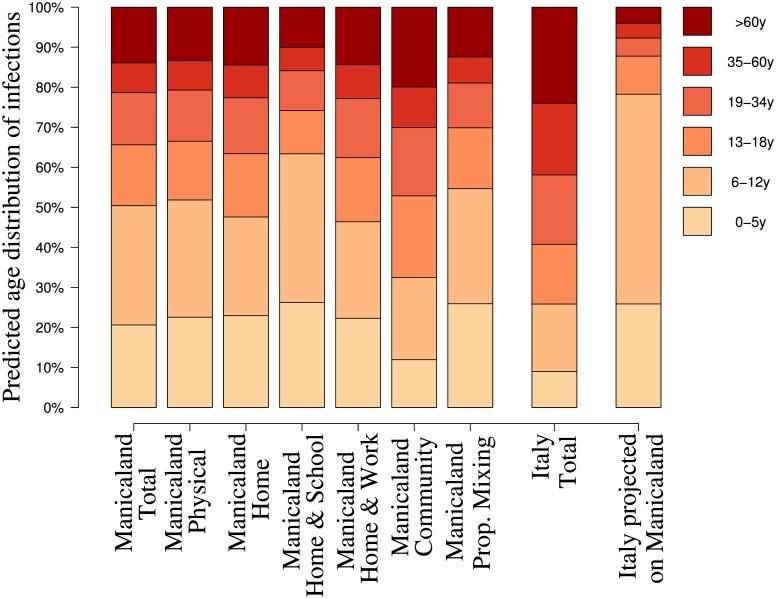
Predicted age distribution of infection cases by type of social contact structure, Manicaland (Zimbabwe), 2013. Seven different social structures derived from the collected data were compared in terms of predicted age distribution of generated infection cases: (from left to right) overall contacts, physical contacts, home contacts, home and school contacts, home and work contacts, general community contacts, proportional mixing based on the average number of contacts in Manicaland and the population of Manicaland. For the sake of comparison, the predicted age distribution of cases is shown also for the overall contact matrix for Italy and for the Italian contact rates applied to the population of Zimbabwe [4].

The predicted age distribution of infected individuals during the exponential phase of an epidemic is highly sensitive to the underlying social contact structure (Fig 5). Our results suggest that, when using the gathered Manicaland data, about 50% of infections are expected to occur among individuals younger than 12 years, while less than 20% among individuals older than 35 years. This pattern is mainly driven by the population age distribution and does not remarkably change when the transmission chain is assumed to be driven by physical contacts only. Similarly, the expected age distribution of cases in the overall population does not change when home contacts are considered alone, suggesting the critical role of the heterogeneous mixing at home for the epidemic transmission chain.

Moreover, our analysis indicates that, when relevant transmission paths are driven by a combination of home and school contacts, school-age children are expected to play a pivotal role in the spread of the infection both within schools and at home. In this case, more than 60% of infections occur among individuals younger than 12 years. On the other hand, when the infection is mainly transmitted through contacts occurring at home and at work, more than one third of the infections are expected among adults and less than 25% among children aged 6–12 years.

Modeling contacts in the general community as driven by proportional mixing leads to overestimating infections in children (mostly pre-school) and underestimating the number of new cases among the elderly. In particular, with proportional mixing, around 55% of infections are predicted to occur among children up to 12 years, whereas, using the collected data on mixing patterns in the general community, only 35% of infections are predicted in the same age group.

The predicted age distribution of cases in Manicaland is found to be considerably different from the one obtained when considering a similar infection process in the Italian population. In fact, when the average contact matrix of Italy is used, more than 20% (versus 13% in Manicaland) of infections are predicted to occur among the elderly and less than 30% (versus 50% in Manicaland) among pre-school and primary school children. Finally, predictions based on the projection of Italian contact rates [4] on the Manicaland age-structure would result in significantly larger estimates of the number of infections among pre-school and primary school children (almost 80% of cases), which suggests the crucial importance of deriving contact patterns in countries characterized by different population age distributions and characteristics of the social structures (households, schools, workplaces, etc.).

## Discussion

In total, 1,245 diaries (corresponding to 26,981 reported contacts) were collected in one rural and one peri-urban township of the Manicaland province of Zimbabwe, with detailed information on individuals’ daily social contacts and use of time. The estimated average number of contacts per person per day is significantly higher in the peri-urban site, and among active students and workers. The derived contact matrix is characterized by a strong age assortativeness among school-aged children and by proportionate mixing at older ages. This pattern is similar to what observed for rural Kenya [17], but differentiates substantially from the European-like mixing [4], which was found to be assortative even among the elderly. This may derive from several factors, among which the younger age distribution of the population in SSA as opposed to European-like settings (in Manicaland, 51% of the population under study was below 20 years of age versus 20% in Italy), the higher proportion of extended families (68% in Manicaland versus 22% in Italy [40]), which leads to large differences in the proportion of home contacts (54% versus 19.7%), and the different use of time (S4 Text). In our study population, during weekdays, individuals reported 63.4% of their daytime at home, 15.3% at school, 1.8% at work, and 19.5% in the general community, as opposed to 57%, 6.2%, 16.7% and 20.1% in Italy, respectively. The proportion of pre-primary school attendance in the age group 0–6 years is extremely low (15% versus 30.7% in Italy), and similarly low is the work participation of adults aged 19–59 years (13.7% versus 26.3% in Italy). Whereas the time at work appeared to be positively associated with the number of overall and work contacts, possibly due to the different work schedules people have, the time spent at school, similar among children, did not result as a determining factor. Conversely, our results suggest that the number of students in the class, rather than the time spent at school, represents the main determinant of the number of school contacts.

Evidence of within-country heterogeneity was also found when comparing the two study sites, with some differences in contact patterns between the rural and the peri-urban site that are worth mentioning. Firstly, due to the different proportions of nuclear and extended families in the two sites, we observed a large number of contacts between the elderly and the younger age groups in the rural area, as well as between parents and their children in the peri-urban area. Secondly, while a higher average number of contacts among school-aged children was found in the rural site, suggesting a possible effect of larger classes, a higher proportion of work contacts was reported for the township.

Interestingly, our modeling simulations have highlighted that the predicted age distribution of infected cases during the exponential phase of an epidemic is highly sensitive to the underlying social contact structure of the study population. In general, when using the gathered Manicaland data, we found that about 50% of infections are expected to occur among individuals aged less than 12 years, and less than 20% among individuals older than 35 years. This result appears robust to the adopted contact definition (all contacts versus physical contacts only). In addition, we found that the heterogeneous nature of home contacts, which is so peculiar of Manicaland data, is the main contributing factor to the transmission chain of an epidemic, and that modeling contacts in the general community as driven by proportional mixing would overestimate the proportion of childhood infections.

A limitation of this study is that data collection did not occur at the same time in both sites, rather it occurred mainly during school holidays in the peri-urban township and during the school term in the subsistence farming area. Even though this procedure possibly led to estimate the number of contacts at school in the township with less accuracy, we do not expect this limitation to have strongly influenced the detection of the qualitative patterns of individual time use and social mixing at school, especially when data from the two sites are pooled together.

Knowledge of social contact patterns is an essential element for a realistic evaluation of the impact of public health control strategies against viral and bacterial infections, such as measles, influenza, tuberculosis, and meningitis, especially in developing countries where vaccination strategies are more difficult to implement and financial constraints are very high. With the current work, we have gathered data on the ways individuals interact in rural and peri-urban areas of SSA and on the factors that mostly facilitate this interaction. We found that the key individual and societal factors contributing to the number of contacts, both overall and in the various settings, are the active participation in school and work, as well as the class size for students. Whereas in the industrialized countries these characteristics may well be captured by individuals’ age, in less developed countries this does not seem to be always the case, as individuals of similar age appeared to have different mixing patterns and thus carrying different risks of transmitting infections. Considering the current demographic transition that SSA countries are undergoing, future increases in the school enrollment and work participation rates may have profound effects on individual interactions. On the other hand, our model results indicate that reductions in household and class sizes, which are also expected to occur as part of the demographic transition, may reduce the overall number of contacts that individuals experience in their daily routine in those two settings. Although the final outcome of these processes is extremely difficult to forecast, the current work highlights which components should be closely monitored to identify possible important changes in social structures affecting individuals’ mixing patterns and infectious disease dynamics. Additional studies in different and distant demographic settings should be considered in order to assess the evolution of these processes over time, and to link specific structures of mixing patterns to relevant individual behaviors and societal characteristics.

## Supporting Information

S1 Dataset Data for the Statistical Analysis. Data on study participants, with summary of the information on social contacts and time use, on which the statistical model is based.
(XLSX)

S2 Dataset Social contact matrix. Social contact matrix *C*_*ij*_ with the estimated (through bivariate smoothing) average number of contacts between participants in age group *i* and contactees in age group *j*, together with the number of individuals in each age group in the reference population.
(XLSX)

S1 Supporting Information Survey Diary. Diary provided to participants aged 6 years or more for the collection of social contacts and time use data.
(PDF)

S1 Text Sampling strategy and study population. Detailed presentation of the study design and of the study population.
(DOCX)

S2 Text Construction of Age-Specific Contact Matrices. Detailed explanation of the construction of the age-specific contact matrices and on the bivariate smoothing technique.
(DOCX)

S3 Text Analysis of Social Contact Data. Detailed explanation of the statistical models for the number of social contacts, and further stratifications of the social contact matrices.
(DOCX)

S4 Text Analysis of Time Use Data. Further stratifications for the time use patterns.
(DOCX)
