# Supplementary material for: Social Contact Structures and Time Use Patterns in the Manicaland Province of Zimbabwe
Source: PLoS One. 2017 Jan 18;12(1):e0170459. doi: 10.1371/journal.pone.0170459 (PMC5242544; doi:10.1371/journal.pone.0170459)
Supplement: S1 Supporting Information — Diary provided to participants aged 6 years or more for the collection of social contacts and time use data. (PDF) [file pone.0170459.s003.pdf]

Exit Interview – To be filled in by researcher

Q1	Did you have a day-off from school/work on the two assigned days?	1° day—Yes	☐	1° day—No	☐
		2° day—Yes	☐	2° day—No	☐
Q2	Did you stay at home because you were ill during the assigned days?	1° day—Yes	☐	1° day—No	☐
		2° day—Yes	☐	2° day—No	☐
Q3	Did you have any difficulties fill-ing in the diary? If YES, please explain the difficulties.	Yes	☐	No	☐
		....			
Q4	Did you record **all** the contacts you had during the period allocated? If NO, explain why.	Yes	☐	No	☐
		....			
Q5	Do you think that the diary changed how you or those around you normally behave? If YES, explain why.	Yes	☐	No	☐
		....			
Q6	Please estimate the age of the person in photographs 1, 2 and 3.	1)	☐	Check	☐
		2)	☐	Check	☐
		3)	☐	Check	☐

Thank you for your participation.

For further questions and comments, kindly contact:

Biomedical Research & Training Institute
Manicaland HIV/STD Prevention Project
Sarupinda Village
Mutasa DC
Tel: 028 2230

MANICALAND HIV/STD PREVENTION PROJECT

CONTACT DIARY

For persons aged 6 years and above

STUDY DETAILS

Q01	ID study participant	Site	
		Household	
		Line	
Q02	Allocated days of the week to fill in the diary		MON/TUE/WED/THU/ FRI/SAT/SUN
Q03	Allocated dates to fill in the diary		DD1-DD2/MM/YYYY
Q04	Person keeping the diary	Participant ☐	
		Shadow(s) ☐	N° of shadows ☐
Q05	Age group participant		

Human diseases are spread through various ways.

One way that some of these infections spread is through coming into contact with infected saliva or mucous released into the air or deposited on hard surfaces through coughing or sneezing.

For us to know exactly how these diseases may spread in the community, we need to know how people of similar or different ages mix as they perform their daily duties.

This diary will enable us to collect data on how people mix in the household and com-munity, and help to understand how to plan for preventive and control measures against the spread of infections.

Diary Checked by:	Initials	Data checked	Signature
Community Researcher			
Team Leader			

INSTRUCTIONS

1	Please keep this diary with you at all times throughout your allocated 48 hour period.
2	**Do not** write your name on this diary. Use only the study identifier assigned to you.
3	Fill in all applicable questions on pages 4-7.
4	Use page 7 to give details of household members only. A household member is anyone who normally eats from the same pot as yourself and slept in the same house as you in the last 24 hours. For each household member only provide the **relationship to you** using the following list:<br>wife/husband; son/daughter; son/daughter in law; grandchild; father/mother; parent -in-law; brother/sister; stepfather/mother; maternal/paternal aunt; maternal/paternal uncle; nephew/niece; cousin; maternal grandfather/mother; paternal grandfather/mother,; adopted/foster child; other relative; not related.
5	On pages 8-9 (DAY 1) and on pages 16-17 (DAY 2), we ask you to report the various places where you have been at different times on the assigned two days and the **number of people present around you at that time and in that place.**
6	On pages 10-15 (DAY 1) and on pages 18-23 (DAY 2), we ask you to record every person you have contact with during the two assigned days, her/his age, and the setting where the contact occurred.
7	If you don't know the exact age, give an estimate of the age, trying to be as accurate as possible.
8	We also ask you whether the contact was physical or non-physical according to the following definition:<br>•a **physical** contact is a skin-to-skin contact such as a handshake or hug (with touching);<br>•a **non-physical** contact is a two-way conversation with three or more words in the physical presence of another person (standing at most at two-arm distance) but no skin-to-skin contact (no touching).

DURING DAY 2, WHO ARE THE PEOPLE YOU ENTERED IN CONTACT WITH?

If you have multiple contacts with a person during the day, record all the settings where you had such a contact and tick "Did you touch?" only if you had at least one physical contact.

"At home" is just for encounters at the residence where you normally live. If you met someone in their home, count this as a "Other" contact.

"At work" stands for encounters that you make at your workplace or throughout the course of your daily job activity (such as meeting customers or clients).

"Other" stands for encounters that you make neither "at home", nor "at school" and nor "at work" (eg. general community, transportation, leisure, church, market, etc).

-->

If you know the exact age of the contact, please specify	Did you touch?	Where did you meet?			
		At home	At school	At work	Other

DURING DAY 2, WHO ARE THE PEOPLE YOU ENTERED IN CONTACT WITH?

In this part of the questionnaire, you should report the different persons you have encountered during the first of two assigned days.

For each person you met during the day, provide a brief description of the person you have encountered (e.g., mother, brother, school-mate, employer, bus driver, friend, etc.)

Remember to record whether it was a skin-to-skin contact, such as a handshake or a hug, or a non-physical contact, that is, a two-way conversation with three or more words in the physical presence of another person (standing at most at two-arm distance), but no skin-to-skin contact.

<—————————————————————————— DAY 2 —

CODE	Relationship of the contact to you	Gender (F/M)	Age						
			< 1	1-5	6-12	13-18	19-34	35-59	60+

INSTRUCTIONS

**9**	Be sure to register each person only once for each day, even if you have several contacts with that person on that day.<br>If you have contact with the same person on both of the days, you should register that person for each of the two days.<br>Each **new** person you contact should have a different line in the diary
**10**	If possible, record each of the your contacts from time-to-time throughout the day to avoid missing any contacts.
**11**	When recording your contacts it is easier to use a **chronological order** according to when you met the person for the first time during your assigned day and then add anyone else that you might remember as you go through your daily activities. However, chronological order is not essential.
**12**	After you have finished recording the diary, we suggest that you double check the diary entries trying to **remember** all of your **activities** to make sure you haven't missed any contact person.
**13**	For the purposes of the study, the day starts at 4 am of the first assigned day, and ends at 4 am of the day following the second assigned day (e.g. if your first assigned day is Monday, you start recording contacts occurred from Monday at 4 am until Wednesday at 4 am).
**14**	**Do not** fill in the last section. A researcher will take you through these questions.
**15**	**Do not** write in any grey areas. These are for administrative purposes only.

STUDY PARTICIPANT DETAILS

**Q06**	Date of birth	DD/MM/YYYY
**Q07**	Including yourself, how many people live in your household?	

SHADOW(S) DETAILS (If not applicable, go directly to Q11)

Q08 Relation of the shadow to the study participant

	1S	2S
Mother		
Teacher		
Other		

If other for 1° shadow, ...

If other for 2° shadow, ...

Q09 Total years of education of the shadow

	1S	2S		1S	2S
0			1-2		
3-7			8-12		
13+			Don't answer		

Q10 Occupation of the shadow

	1S	2S		1S	2S
Teacher			Office worker		
Agriculture/ fishing			Retail (shops, petty trading)		
Casual labour			Retired		
Unemployed			Other		

If other for 1° shadow, ...

If other for 2° shadow, ...

DURING DAY 2, WHO ARE THE PEOPLE YOU ENTERED IN CONTACT WITH?

If you have multiple contacts with a person during the day, record all the settings where you had such a contact and tick "Did you touch?" only if you had at least one physical contact.

"At home" is just for encounters at the residence where you normally live. If you met someone in their home, count this as a "Other" contact.

"At work" stands for encounters that you make at your workplace or throughout the course of your daily job activity (such as meeting customers or clients).

"Other" stands for encounters that you make neither "at home", nor "at school" and nor "at work" (eg. general community, transportation, leisure, church, market, etc).

———————————————————————————>

If you know the exact age of the contact, please specify	Did you touch?	Where did you meet?			
		At home	At school	At work	Other

DURING DAY 2, WHO ARE THE PEOPLE YOU ENTERED IN CONTACT WITH?

In this part of the questionnaire, you should report the different persons you have encountered during the first of two assigned days.

For each person you met during the day, provide a brief description of the person you have encountered (e.g., mother, brother, school-mate, employer, bus driver, friend, etc.)

Remember to record whether it was a skin-to-skin contact, such as a handshake or a hug, or a non-physical contact, that is, a two-way conversation with three or more words in the physical presence of another person (standing at most at two-arm distance), but no skin-to-skin contact.

<———————————————————————————— DAY 2 —

CODE	Relationship of the contact to you	Gender (F/M)	Age						
			< 1	1-5	6-12	13-18	19-34	35-59	60+

SCHOOL DETAILS OF THE STUDY PARTICIPANT IF APPLICABLE

**Q11**	Are you currently attending school?	Yes<br>No	☐<br>☐	**If no, go directly to Q19**	
**Q12**	What level are you in?	Pre-school<br>Secondary	☐<br>☐	Primary<br>College/University	☐<br>☐
**Q13**	What is the name of the school you are currently attending?		☐		
**Q14**	Where is the school located?	Village/ town<br>District	☐<br>☐		
**Q15**	Estimate the distance from home to your school.		☐ **km**		
**Q16**	How do you get to school?	On foot<br>By bike<br>Other	☐<br>☐<br>☐	By bus<br>By car<br>If other, ………………………………	☐<br>☐
**Q17**	How many students are attending your school?		☐		
**Q18**	How many students are in the class where you spend most of your time?		☐		

WORK DETAILS OF THE STUDY PARTICIPANT IF APPLICABLE

**Q19**	Have you ever worked to earn money outside of the house-hold?	Yes ☐<br>No ☐	**If no, go directly to Q25**
**Q20**	Which sector of employment are you currently working in?<br><br>**If unemployed or retired,**	Teacher ☐<br>Agriculture/ fishing ☐<br>Casual labour ☐<br>Unemployed ☐	Office worker ☐<br>Retail (shops, petty trade) ☐<br>Retired ☐<br>Other ☐<br>If other, ...
**Q21**	Where is your workplace lo-cated?	Village/town<br>District	
**Q22**	Estimate the distance from home to your workplace.		**km**
**Q23**	How do you get to work?	On foot ☐<br>By bike ☐<br>Other ☐	By bus ☐<br>By car ☐<br>If other, ...
**Q24**	Approximately how many people are employed in your workplace (incl. yourself)?		

DURING DAY 2, WHO ARE THE PEOPLE YOU ENTERED IN CONTACT WITH?

If you have multiple contacts with a person during the day, record all the settings where you had such a contact and tick "Did you touch?" only if you had at least one physical contact.

"At home" is just for encounters at the residence where you normally live. If you met someone in their home, count this as a "Other" contact.

"At work" stands for encounters that you make at your workplace or throughout the course of your daily job activity (such as meeting customers or clients).

"Other" stands for encounters that you make neither "at home", nor "at school" and nor "at work" (eg. general community, transportation, leisure, church, market, etc).

--->

If you know the exact age of the contact, please specify	Did you touch?	Where did you meet?			
		At home	At school	At work	Other

DURING DAY 2, WHO ARE THE PEOPLE YOU ENTERED IN CONTACT WITH?

In this part of the questionnaire, you should report the different persons you have encountered during the first of two assigned days.

For each person you met during the day, provide a brief description of the person you have encountered (e.g., mother, brother, school-mate, employer, bus driver, friend, etc.)

Remember to record whether it was a skin-to-skin contact, such as a handshake or a hug, or a non-physical contact, that is, a two-way conversation with three or more words in the physical presence of another person (standing at most at two-arm distance), but no skin-to-skin contact.

<——————————————————————————— DAY 2 —

CODE	Relationship of the contact to you	Gender (F/M)	Age						
			< 1	1-5	6-12	13-18	19-34	35-59	60+

HOUSEHOLD DETAILS OF THE STUDY PARTICIPANT

Q25 A household member is anyone who normally eats from the same pot as yourself and slept in the same house as you in the last 24 hours.

List all the different people who belong to your household based on the relationship this person has with you (see detailed list on page 2)

CODE	Relationship to you	Age (years)	Gender (F/M)	Do you sleep in the same room? (Y/N)

DURING DAY 1, WHERE DID YOU SPEND YOUR TIME?

In this part of the questionnaire, we ask you to report the various settings where you have been during day 1 of the two assigned days and the number of people present in that setting.

If in a certain time slot you have been in different settings, tick a box for each of the settings that apply.

DURING DAY 2, WHERE DID YOU SPEND YOUR TIME?

"At home" means the residence where you normally live. If you spent time in someone else's home the "Other" setting category applies.

"At work" can either mean at your workplace or throughout the course of your daily job activity (such as meeting customers or clients).

"Other" refers to settings different from "at home", "at school", and "at work" (eg. general community, transportation, leisure, church, market, etc).

For each of the settings you have been in, report the ***number of people present around you at that time****. If this is difficult, please provide an estimate.*

<——————————————————————————DAY 1 ——————————————————————————————>

Time slots	Where were you at that time?							How many people were present around you at that time, including yourself?			
	At home	At school	At work	Other				At home	At school	At work	Other
				Within the Village	Within the Village, outside the homestead	Within the District, outside the Village	Outside the District				
Dawn (approx. 04:00-05:00)											
Twilight (approx.5:00-06:00)											
Sunrise (approx. 6:00-7:00)											
Early morning (7:00-10:00)											
Late morning (10:00-12:00)											
Midday/Lunch (12:00-14:00)											
Early afternoon (14:00-16:00)											
Late afternoon (16:00-17:00)											
Sunset (approx. 17:00-18:00)											
Twilight (approx. 18:00-19:00)											
Dusk (approx. 19:00-20:00)											
Early night (20:00-0:00)											
Late night (0:00-4:00)											

DURING DAY 2, WHERE DID YOU SPEND YOUR TIME?

In this part of the questionnaire, we ask you to report the various settings where you have been during day 2 of the two assigned days and the number of people present in that setting.

If in a certain time slot you have been in different settings, tick a box for each of the settings that apply.

DURING DAY 1, WHERE DID YOU SPEND YOUR TIME?

"At home" means the residence where you normally live. If you spent time in someone else's home the "Other" setting category applies.

"At work" can either mean at your workplace or throughout the course of your daily job activity (such as meeting customers or clients).

"Other" refers to settings different from "at home", "at school", and "at work" (eg. general community, transportation, leisure, church, market, etc).

For each of the settings you have been in, report the ***number of people present around you at that time****. If this is difficult, please provide an estimate.*

<——————————————————————————DAY 2 ————————————————————————————————>

Time slots	Where were you at that time?							How many people were present around you at that time, including yourself?			
	At home	At school	At work	Other				At home	At school	At work	Other
				Within the Village	Within the Ward, outside the Village	Within the District, outside the Ward	Outside the District				
**Dawn (approx. 04:00-05:00)**											
**Twilight (approx.5:00-06:00)**											
**Sunrise (approx. 6:00-7:00)**											
**Early morning (7:00-10:00)**											
**Late morning (10:00-12:00)**											
**Midday/Lunch (12:00-14:00)**											
**Early afternoon (14:00-16:00)**											
**Late afternoon (16:00-17:00)**											
**Sunset (approx. 17:00-18:00)**											
**Twilight (approx. 18:00-19:00)**											
**Dusk (approx. 19:00-20:00)**											
**Early night (20:00-0:00)**											
**Late night (0:00-4:00)**											

DURING DAY 1, WHO ARE THE PEOPLE YOU ENTERED IN CONTACT WITH?

In this part of the questionnaire, you should report the different persons you have encountered during the first of two assigned days.

For each person you met during the day, provide a brief description of the person you have encountered (e.g., mother, brother, school-mate, employer, bus driver, friend, etc.)

Remember to record whether it was a skin-to-skin contact, such as a handshake or a hug, or a non-physical contact, that is, a two-way conversation with three or more words in the physical presence of another person (standing at most at two-arm distance), but no skin-to-skin contact.

DURING DAY 1, WHO ARE THE PEOPLE YOU ENTERED IN CONTACT WITH?

If you have multiple contacts with a person during the day, record all the settings where you had such a contact and tick "Did you touch?" only if you had at least one physical contact.

"At home" is just for encounters at the residence where you normally live. If you met someone in their home, count this as a "Other" contact.

"At work" stands for encounters that you make at your workplace or throughout the course of your daily job activity (such as meeting customers or clients).

“Other” stands for encounters that you make neither “at home”, nor “at school” and nor “at work” (eg. general community, transportation, leisure, church, market, etc).

<—————————————————————— DAY 1 ——————————————————————>

CODE	Relationship of the contact to you	Gender (F/M)	Age							If you know the exact age of the contact, please specify	Did you touch?	Where did you meet?			
			< 1	1-5	6-12	13-18	19-34	35-59	60+			At home	At school	At work	Other

DURING DAY 1, WHO ARE THE PEOPLE YOU ENTERED IN CONTACT WITH?

In this part of the questionnaire, you should report the different persons you have encountered during the first of two assigned days.

For each person you met during the day, provide a brief description of the person you have encountered (e.g., mother, brother, school-mate, employer, bus driver, friend, etc.)

Remember to record whether it was a skin-to-skin contact, such as a handshake or a hug, or a non-physical contact, that is, a two-way conversation with three or more words in the physical presence of another person (standing at most at two-arm distance), but no skin-to-skin contact.

DURING DAY 1, WHO ARE THE PEOPLE YOU ENTERED IN CONTACT WITH?

If you have multiple contacts with a person during the day, record all the settings where you had such a contact and tick "Did you touch?" only if you had at least one physical contact.

"At home" is just for encounters at the residence where you normally live. If you met someone in their home, count this as a "Other" contact.

"At work" stands for encounters that you make at your workplace or throughout the course of your daily job activity (such as meeting customers or clients).

“Other” stands for encounters that you make neither “at home”, nor “at school” and nor “at work” (eg. general community, transportation, leisure, church, market, etc).

<——————————————— DAY 1 ———————————————>

CODE	Relationship of the contact to you	Gender (F/M)	Age							If you know the exact age of the contact, please specify	Did you touch?	Where did you meet?			
			< 1	1-5	6-12	13-18	19-34	35-59	60+			At home	At school	At work	Other

DURING DAY 1, WHO ARE THE PEOPLE YOU ENTERED IN CONTACT WITH?

In this part of the questionnaire, you should report the different persons you have encountered during the first of two assigned days.

For each person you met during the day, provide a brief description of the person you have encountered (e.g., mother, brother, school-mate, employer, bus driver, friend, etc.)

Remember to record whether it was a skin-to-skin contact, such as a handshake or a hug, or a non-physical contact, that is, a two-way conversation with three or more words in the physical presence of another person (standing at most at two-arm distance), but no skin-to-skin contact.

DURING DAY 1, WHO ARE THE PEOPLE YOU ENTERED IN CONTACT WITH?

If you have multiple contacts with a person during the day, record all the settings where you had such a contact and tick "Did you touch?" only if you had at least one physical contact.

"At home" is just for encounters at the residence where you normally live. If you met someone in their home, count this as a "Other" contact.

"At work" stands for encounters that you make at your workplace or throughout the course of your daily job activity (such as meeting customers or clients).

“Other” stands for encounters that you make neither “at home”, nor “at school” and nor “at work” (eg. general community, transportation, leisure, church, market, etc).

<———————————————————— DAY 1 ————————————————————>

CODE	Relationship of the contact to you	Gender (F/M)	Age							If you know the exact age of the contact, please specify	Did you touch?	Where did you meet?			
			< 1	1-5	6-12	13-18	19-34	35-59	60+			At home	At school	At work	Other
