# Supplementary material for: Social Contact Structures and Time Use Patterns in the Manicaland Province of Zimbabwe
Source: PLoS One. 2017 Jan 18;12(1):e0170459. doi: 10.1371/journal.pone.0170459 (PMC5242544; doi:10.1371/journal.pone.0170459)
Supplement: S1 Text — Detailed presentation of the study design and of the study population. (DOCX) [file pone.0170459.s004.docx]

S1 Text

**Sampling strategy and study population**

**Study design**

The study is a cross-sectional survey with stratified random sampling by age group and site. Seven age groups were used for sampling purposes: infants (<1 year), preschool (1–5 years), primary school children (6–12 years), secondary school children (13–18 years), young adults (19–34 years), older adults (35–60 years), and the elderly (> 60 years).

Two sites, out of the twelve involved in the Manicaland HIV/STD Prevention Study [1], were selected, namely, a peri-urban township and a subsistence farming area. For practical reasons, and in line with the Manicaland HIV/STD Prevention Study, each site was further divided into three areas, each one served by a different team of field researchers and designed to be as homogeneous as possible in terms of urban/rural type of setting, to have roughly the same number of households, and to be geographically adjacent.

The target population of the contact study was formed by individuals of all ages living in the two sites. In particular, all people living in households that were selected in the previous five rounds of the Manicaland HIV/STD Prevention Study, as well as individuals living in (new) households registered only during the current sixth round, were considered eligible for inclusion in the study. We also included in the study children from birth to 2 years old and people older than 54 years old, as these age groups were not considered in the Manicaland HIV/STD Prevention Study (only people aged 15–54 have been included since the first round and children aged at least 2 years old have only been included from the fifth round). Exceptions to this general rule were allowed only when there were not enough individuals in the population to fulfil the sampling requirements and, in particular, the sample size for each of the defined age strata. In such a case, the remaining individuals were sampled from the whole population of the area/site (independently from being included or not in the study population of the Manicaland HIV/STD Prevention Study).

More practically, inclusion criteria for the study were 1) being a permanent resident or regular visitor of the household (HH) in either of the two sites, and 2) giving written individual informed consent (from a parent/guardian if participant was below 18 years of age). Exclusion criteria were 1) refusing to consent, and 2) planning to move out of the site within two weeks of the first visit of the field researcher.

In each of the two sites, each cluster was assigned a constant quota of questionnaires to be filled in, out of the total target number of planned 600 questionnaires per site (1200 questionnaires in total). The sample allocation was stratified by the following two variables: 1) age group (< 1, 1–5, 6–12, 13–18, 19–34, 35–60, > 60) and 2) site (rural and peri-urban).

As it can be inferred from the chosen age groups, we were mostly interested in the contact patterns and the use of time of children, who are the main spreaders of childhood infections. The sample allocation, i.e., the number of required samples per age group and per site, is presented in Table A, together with the census/sample (C/S) ratio per age group, and the frequency of that age group in the peri-urban and rural population, respectively. If the C/S ratio for an age group is smaller than one, it means that we are oversampling in that age group; on the contrary, if the C/S ratio is larger than one, then we are under sampling from that age group. In the first four age groups, the sample was allocated to have at least a power of 50%, 80%, 80%, and 50% in each age group, respectively. For the remaining age groups, we divided what remained of the target sample size proportionally to the weight of the age groups in the rural and in the peri-urban population.

In general, in each household only one person was selected to take part in the study. In order to allocate faster the sample by age, we used a set of priorities inversely proportional to the age distribution of the population in each site (see Table B), so that the less frequent an age group in the population, the higher the priority for its selection. Each selected person was assigned a personal identifier (ID), which was constructed as the combination of the codes for the site, the household, and a person identifier within the household. In such a way, each participant had the same ID in both the Manicaland HIV/STD Prevention Study and the contact study.

Individual consent was sought, after providing a detailed explanation of the study. In particular, during consenting, participants were informed that they would have had to keep one diary for 48 consecutive hours, starting on a randomly allocated first day. To select the days, field researchers used a set of seven cards, one per each day of the week. The participant randomly picked one card, corresponding to a specific day, and then the assigned two-day period consisted on that day plus the following day of the week. For example, if a person drew the "Saturday" card, that person would have been assigned to the coming Saturday and Sunday.

The exception to the rule "one household, one sampled person" occurred only when the person selected in the household was a child aged less than 1 year. In such a case, the field researcher selected a second person in the household to be interviewed (still according to the priorities listed in Table B). After removing the two cards corresponding to the two-days period assigned to the first person, the second selected person drew a card from the remaining ones. In this way, the two participants had to fill in their own diaries in two different, not overlapping, periods. If in one household there were more than one eligible person, e.g., two or three members belonging to the same age group that was chosen according to the list of priorities, then the participant was randomly selected giving to each eligible person an equal inclusion probability. Therefore, if there were two eligible persons, each had a probability of 50% to be selected.

Sampling weights – the inverse of the probability that an observation is included because of the sampling design - were calculated for each site separately, based on age census data collected in 2013 in the two sites by the Manicaland HIV/STD Prevention Study and used to compute site-specific estimates. Moreover, population weights – the ratio between the site’s population and the site’s sample size – were used to compute overall statistics. These overall statistics should be considered indicative of general trends and levels of the Manicaland province, but specific statistical representativeness for the whole of Zimbabwe is not claimed.

Table A. Sample allocation. Sample allocation by site and age group, with C/S ratio and relative age distribution of the population.

| **Peri-urban township** | | | | **Subsistence farming area** | | | |
| --- | --- | --- | --- | --- | --- | --- | --- |
| **Age group** | **N** | **C/S** | **Pop. (%)** | **Age group** | **N** | **C/S** | **Pop. (%)** |
| <1 | 61 | 0.31 | 3.1% | <1 | 61 | 0.36 | 3.7% |
| 1–5 | 125 | 0.57 | 11.9% | 1–5 | 125 | 0.69 | 14.5% |
| 6–12 | 125 | 0.72 | 15.0% | 6–12 | 139 | 0.95 | 22.0% |
| 13–18 | 80 | 0.95 | 12.7% | 13–18 | 89 | 0.95 | 14.1% |
| 19–34 | 123 | 1.64 | 33.7% | 19–34 | 88 | 1.48 | 21.7% |
| 35 - 60 | 72 | 1.64 | 19.7% | 35 - 60 | 67 | 1.48 | 16.5% |
| >60 | 14 | 1.64 | 3.9% | >60 | 31 | 1.48 | 7.7% |

Table B. Age distribution in the two sites. Age groups in two sites sorted by priority to be given, from the highest to the lowest, based on the age distribution of the whole Zimbabwean population in 2013.

| **Peri-urban township** | | **Subsistence farming area** | |
| --- | --- | --- | --- |
| **Age group** | **Urban population (%)** | **Age group** | **Rural population (%)** |
| < 1 | 3.12% | < 1 | 3.68% |
| >60 | 3.88% | > 60 | 7.74% |
| 1–5 | 11.94% | 13–18 | 14.09% |
| 13–18 | 12.66% | 1–5 | 14.45% |
| 6–12 | 14.98% | 35–60 | 16.45% |
| 35–60 | 19.72% | 19–34 | 21.65% |
| 19–34 | 33.69% | 6–12 | 21.95% |

**Study population**

In what follows, we show the demographic characteristics of the study population in terms of age distribution of the population, and household size distribution. Moreover, we also show the demographic characteristics of the collected sample in terms of age and household size distribution, proportion of people living in nuclear families, and age distribution of household heads between peri-urban and rural sites and between nuclear and extended families.

It is important to highlight that the rural study site can be considered as representative of rural Zimbabwe [2]. In particular, the percentage of individuals aged 19-59 yrs. in the population of the rural site is 36.04%, while in rural Zimbabwe is 34.42%. Instead, as regards the percentage of households with 6 or more members, in the study site we found a value of 27.34%, and in rural Zimbabwe of 28%.

On the other hand, the peri-urban site can be considered as illustrative of a *rural–urban* transition zone. Specifically, the percentage of individuals aged 19-59 yrs. in the population of the peri-urban site is 40.77%, while in urban Zimbabwe is 48.56%. Instead, as regards the percentage of households with 6 or more members, in the study site we found a value of 19.82%, and in urban Zimbabwe of 19%.

Fig A. Population age distributions. Comparison of population’s age distribution: a) Zimbabwe (US Census data, 2013), b) Two study sites pooled together (Manicaland HIV/STD Prevention Study Census data, 2013), and c) Italy (US Census data, 2003).


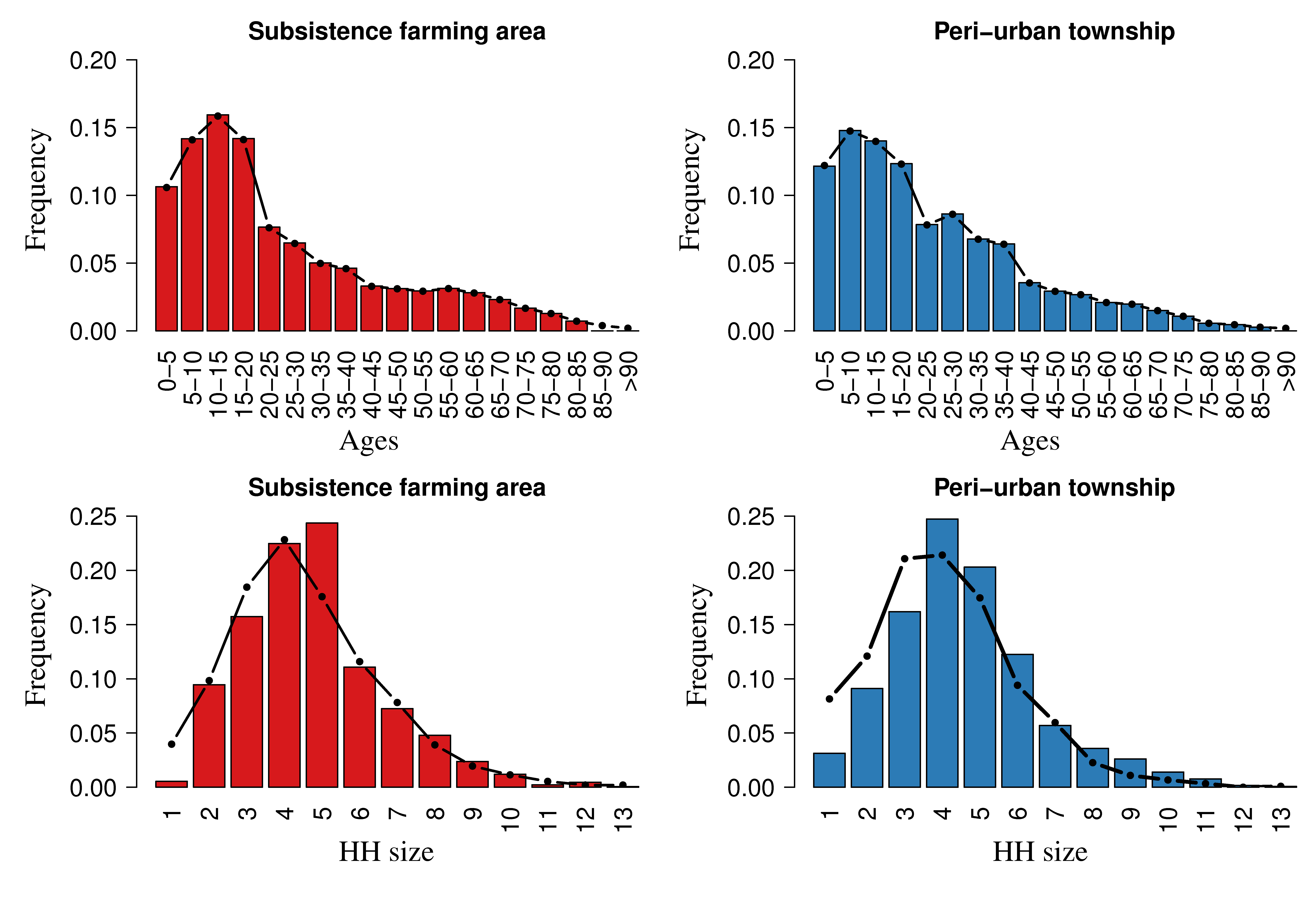


Fig B. Age distribution and HH size by site. Demographics of the two sites, comparing the sample (bar plot) with the census data from the sites (lines) for the subsistence farming area (left side, in red) and the peri-urban township (right side, in blue).


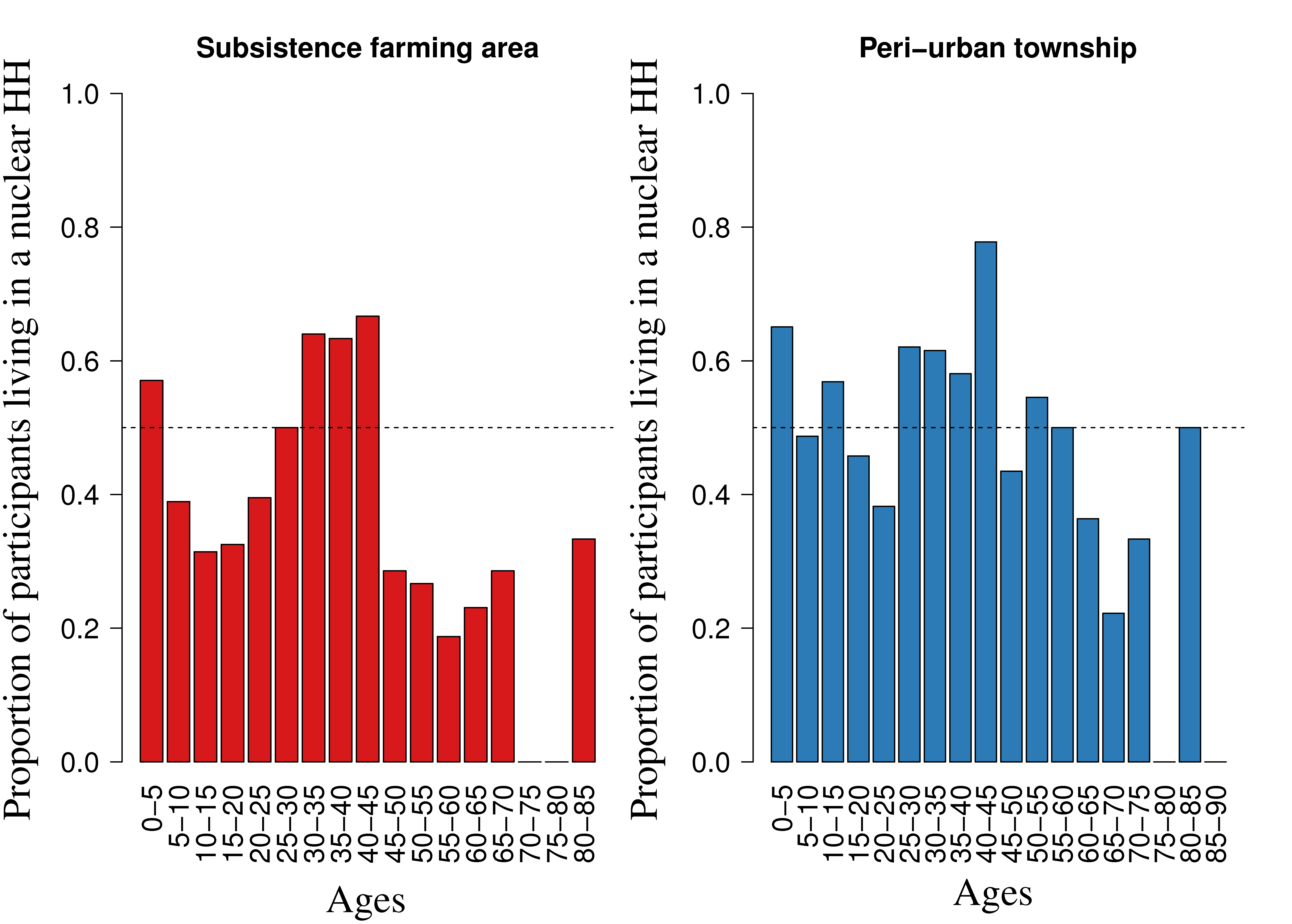


Fig C. Proportion of participants living in nuclear HHs, by site. Bar plot of the proportion of participants living in a nuclear household, in the subsistence farming area (left panel), and in the peri-urban township (right panel). The horizontal dashed line indicates a proportion of 0.5.


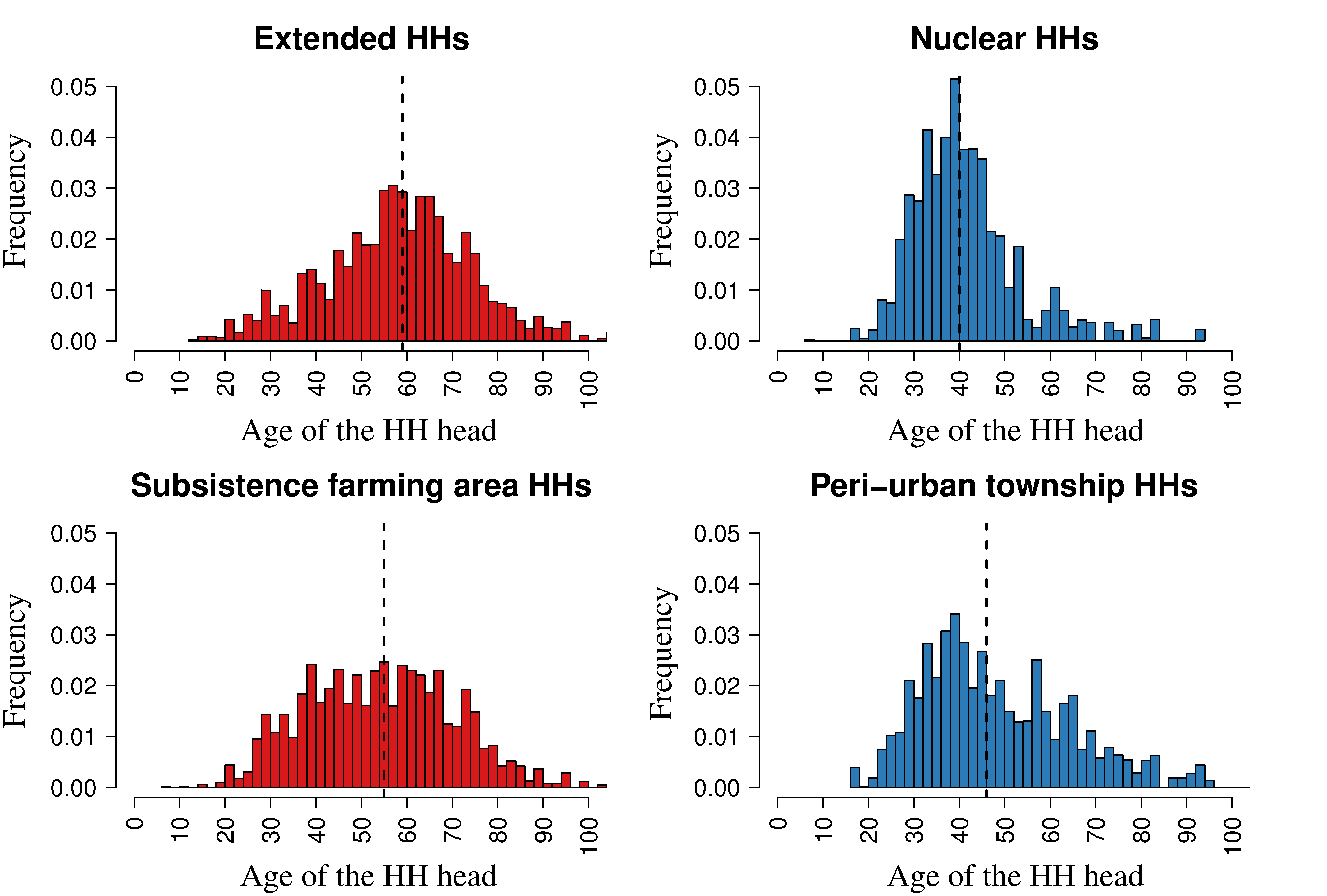


Fig D. Age distribution of HH heads, by site and by HH type. Histogram of the distribution of the age of the household heads, in extended and nuclear households (top row), and in the subsistence farming area and in the peri-urban township (bottom row). The vertical dashed line in each graph represents the median age of the household head per setting.

**Reference**

1. Gregson S, Garnett GP, Nyamukapa CA, Hallett TB, Lewis JJC, Mason PR, et al. HIV Decline Associated with Behavior Change in Eastern Zimbabwe. Science. 2006;311: 664–666. doi:10.1126/science.1121054

2. Zimbabwe National Statistics Agency, ICF International. Zimbabwe Demographic and Health Survey 2010-11. Calverton, MA: Zimbabwe National Statistics Agency (ZIMSTAT) and ICF International; 2012 Mar.
