# Supplementary material for: Social Contact Structures and Time Use Patterns in the Manicaland Province of Zimbabwe
Source: PLoS One. 2017 Jan 18;12(1):e0170459. doi: 10.1371/journal.pone.0170459 (PMC5242544; doi:10.1371/journal.pone.0170459)
Supplement: S2 Text — Detailed explanation of the construction of the age-specific contact matrices and on the bivariate smoothing technique. (DOCX) [file pone.0170459.s005.docx]

S2 Text

**Construction of Age-Specific Contact Matrices**

Using the collected social contact data, we primarily aimed at estimating the age-specific contact rate per person per day. Let indices $i$ and $j$ represent the age, such that $i,j=1,2,\ldots,16$, corresponding to 5-year age groups up to 79 years of age. Further, let $n_{i}$ be the total number of participants in age group $i$ such that $\sum_{i=1}^{16} n_{i}=n$, the total number of participants in the study. Let $y_{ij,k}$ be the number of contacts that participant $k$ in age group $i$ has with contacts in age group $j$. Then, the total number of observed contacts, denoted $M_{\mathrm{ij}}$, is given by

$M_{\mathrm{ij}}=\sum_{k=1}^{n_{i}} y_{ij,k}.$ (1)

In order to compute the average number of observed contacts per person per day, we need to account for the fact that the sample age distribution is different from the population age distribution by using sampling weights. As already mentioned in the main text, sample weights, denoted by $w_{i,sd,k}$, are given by the reciprocal of the probability $p_{i,sd,k}$ of an individual to be included in the sample:

$w_{i,sd,k}=\frac{1}{p_{i,sd,k}},$ (2)

where $i$ denotes the age group, $s$ denotes the site (urban or rural), $d$ denotes the day of the survey (day 1 or day 2), and the probability $p_{i,sd,k}$ is given by

$p_{i,sd,k}=\frac{n_{i,sd}}{N_{i,sd}},$ (3)

where $n$ stands for the sample size, and $N$ stands for the study population in Manicaland, as obtained from the census data gathered during the sixth wave of the Manicaland HIV/STD Prevention Study. Given the individual sample weights $w_{i,sd,k}$, the average number of observed contacts is given by the following formula:

$m_{ij,sd}=\frac{\sum_{k=1}^{n_{i,sd}} y_{ij,sd,k}w_{i,sd,k}}{\sum_{k=1}^{n_{i,sd}} w_{i,sd,k}}.$ (4)

In this particular case, since sampling weights are specific per age group, site, and day, that is to say, $w_{i,sd,k}=w_{i,sd}$, then Eq. (4) simplifies in the following way:

$m_{ij,sd}=\frac{\sum_{k=1}^{n_{i,sd}} y_{ij,sd,k}w_{i,sd}}{\sum_{k=1}^{n_{i,sd}} w_{i,sd}}$ (5)

$=\frac{w_{i,sd}\sum_{k=1}^{n_{i,sd}} y_{ij,sd,k}}{n_{i,sd}w_{i,sd}}$ (6)

$=\frac{\sum_{k=1}^{n_{i,sd}} y_{ij,sd,k}}{n_{i,sd}}.$ (7)

If the weights were calculated based on further variables, for instance, by distinguishing by household size, then we should have used those presented in Eq. (4).

To obtain the matrix with the average number of observed contacts in the two sites (over the two days) or in the two days (over the two sites), it is possible to do it by weighting the site-and-day-specific matrices by the sample sizes per day of the survey and per site, respectively:

$m_{ij,s}=\frac{\sum_{d=1}^{2} m_{ij,sd}n_{i,s,d}}{\sum_{d=1}^{2} n_{i,s,d}}\mathrm{and}m_{ij,d}=\frac{\sum_{s=1}^{2} m_{ij,sd}n_{i,d,s}}{\sum_{s=1}^{2} n_{i,d,s}}.$ (8)

Finally, the matrix containing the overall mean number of observed contacts can be obtained using either $m_{ij,s}$ or $m_{ij,d}$ with a final weighted mean between matrices:

$m_{\mathrm{ij}}=\frac{\sum_{s=1}^{2} m_{ij,s}n_{i,s}}{\sum_{s=1}^{2} n_{i,s}}\mathrm{or}m_{\mathrm{ij}}=\frac{\sum_{d=1}^{2} m_{ij,d}n_{i,d}}{\sum_{d=1}^{2} n_{i,d}}.$ (9)

Once the matrix containing the mean number of contacts per person per day $m_{\mathrm{ij}}$ is available, it is possible to compute the age-specific contact rates through a series of straightforward steps. First, we need to estimate the expected number of contacts at the population level, denoted as $C_{\mathrm{ij}}$, by multiplying the matrix $m_{\mathrm{ij}}$ by the age distribution of the population $\mathrm{po}p_{i}$:

$C_{\mathrm{ij}}=m_{\mathrm{ij}}\times pop_{i}.$ (10)

Second, we must take into account that, at the population level, it is required for contacts to be reciprocal, that is to say, if individual A is meeting individual B, then also individual B is meeting individual A. This means that $m_{\mathrm{ij}}\mathrm{po}p_{i}=m_{\mathrm{ji}}\mathrm{po}p_{j}$ or, that is the same, $C_{\mathrm{ij}}=C_{\mathrm{ji}}$. Hence, in order to correct for reciprocity of contacts, the elements of the symmetric matrix at population level $Cs_{\mathrm{ij}}$ are obtained in the following way:

$Cs_{\mathrm{ij}}=\frac{C_{\mathrm{ij}}n_{i}+C_{\mathrm{ji}}n_{j}}{n_{i}+n_{j}},$ (11)

where the elements of the population matrix $C_{\mathrm{ij}}$ and $C_{\mathrm{ji}}$ are weighted for the $n_{i}$ and $n_{j}$, that is, the age group size of participants and contacts in the sample, respectively.

Then, we obtain the average number of contacts between an individual aged $i$ with contacts aged $j$ by dividing the matrix $\mathrm{Cs}$ for the age distribution of the population from which participants are drawn:

$ms_{\mathrm{ij}}=\frac{Cs_{\mathrm{ij}}}{\mathrm{po}p_{i}}.$ (12)

At last, we can compute the contact rates at which an individual aged $i$ contacts per day an individual aged $j$ from the matrix with the average number of contacts, corrected by reciprocity, $\mathrm{ms}$, by dividing for the age distribution of the population from which contacts are drawn:

$cs_{\mathrm{ij}}=\frac{ms_{\mathrm{ij}}}{\mathrm{po}p_{j}}.$ (13)

As regards the age of respondents and their contactees, we considered the exact ages given by respondents and we grouped them into 5-year age groups until 79 years of age. As respondents were asked to give the age group of contactee and to add the exact age only if known or easily estimable, a proportion of contacts will be missing the exact age. To solve this issue, we implemented a multiple imputation approach [1]. For each contactee with missing information, we imputed the missing age by drawing it randomly, with replacement, from the age group of the contactee, proportionally to the age distribution in the population. In this way, we imputed the missing ages for 20 times, generating therefore 20 complete data sets. For each data set, a matrix with the observed number of contacts between age group *i* and age group *j* was obtained. Finally, the 20 matrices were combined together through the simple mean to obtain a single matrix with the observed number of contacts between age groups.

**Bivariate smoothing for contact matrices**

We used bivariate smoothing in order to estimate the $c_{ij}$ elements of the social contact matrix [2-5]. The expected number of contacts between person aged $i$ with person aged $j$ is modeled using a two-dimensional continuous function applied to the age of participants and respondents, respectively, via a generalized additive model (GAM) [2]. We used smooth of both age variables from tensor products to provide more flexibility to the fit. To allow for over dispersion in the number of contacts, we assumed that they are distributed according to a negative binomial distribution with mean $c_{ij}$, dispersion parameter $k$, and variance $c_{ij}+{c_{ij}^{2}}/k$. We chose the basis dimension $K$=10 in order to be sufficiently large to fit the data well, while keeping the fitting procedure computationally efficient [2]. Then, we chose as smoothing base the thin plate regression splines, in order to avoid the selection of knots, and a log link function for the GAM, which also included an offset term for the number of participants in age group $i$. Finally, we applied a smooth-then-constrain approach [4] to account for the reciprocal nature of data, after having projected the expected number of contacts at the population level.

**References**

1. Rubin DB. Multiple Imputation for Nonresponse in Surveys. Rubin DB, editor. Hoboken, NJ, USA: John Wiley & Sons; 1987. doi:10.1002/9780470316696

2. Wood S. Generalized Additive Models. CRC Press; 2006.

3. Hens N, Goeyvaerts N, Aerts M, Shkedy Z, Van Damme P, Beutels P. Mining social mixing patterns for infectious disease models based on a two-day population survey in Belgium. BMC Infect Dis. 2009;9: 5. doi:10.1186/1471-2334-9-5

4. Goeyvaerts N, Hens N, Ogunjimi B, Aerts M, Shkedy Z, Van Damme P, et al. Estimating infectious disease parameters from data on social contacts and serological status. Appl Statist. 2010;59: 255–277. doi:10.1111/j.1467-9876.2009.00693.x

5. Hens N, Shkedy Z, Aerts M, Faes C, Van Damme P, Beutels P. Modeling Infectious Disease Parameters Based on Serological and Social Contact Data. 1st ed. Gail M, Krickeberg K, Sarnet J, Tsiatis A, Wong W, editors. New York, NY: Springer; 2012. pp. 1–299.
