# Supplementary material for: Social Contact Structures and Time Use Patterns in the Manicaland Province of Zimbabwe
Source: PLoS One. 2017 Jan 18;12(1):e0170459. doi: 10.1371/journal.pone.0170459 (PMC5242544; doi:10.1371/journal.pone.0170459)
Supplement: S3 Text — Detailed explanation of the statistical models for the number of social contacts, and further stratifications of the social contact matrices. (DOCX) [file pone.0170459.s006.docx]

S3 Text

**Analysis of Social Contact Data**

**Statistical modelling of social contact data**

Generalized estimating equations (GEEs) [1] are used to statistically model both the overall number of social contacts and the setting-specific number of social contacts of study participants, in order to identify their main determinants. In particular, GEEs estimate the marginal effect of determinants, while accounting for the individual-specific correlation between the number of contacts over the two survey days, and adjusting for all the other possible explanatory variables. We assumed a negative binomial distribution for the number of contacts, in order to account for over dispersion [2], and a constant correlation between contacts over the two survey days. Among the socio-demographic explicatory variables, we used a measure of socio-economic status (SES) for the household of participants. This SES index, which is reported in Table A, is calculated using data on household characteristics and owned assets [3] available from the Manicaland HIV/STD Prevention Study dataset [4]. In particular, respondents were asked to provide information on the main source of drinking water in the household, on the kind of toilet facility, whether the toilet facility was used only by household members or was rather shared with neighbors or communal, on the owned assets (electricity, refrigerator, radio, television, bicycle, motorcycle, car, and tractor), on the type of household, and on the type of floor of the main dwelling. All these variables were reported as ordered categorical variables, with higher values implying a higher socio-economic status, and were given as input of a principal component analysis. The SES index was extracted as the first principal component, which accounted for 32% of the total variability in the household asset data.

**Table A** **Number of social contact by site and socio-demographic characteristics.** Total, median and IQR of the number of reported contacts per person/day by site and by additional stratifications. The p-value for the Wilcoxon test for each category between the two sites is also reported.

| Variable | Category | Peri-urban township | | Subsistence farming area | | *P-value* |
| --- | --- | --- | --- | --- | --- | --- |
|  |  | **Person-days** | **Median (IQR)** | **Person-days** | **Median (IQR)** |  |
| Overall |  | 1108 | 10 (6-14) | 1382 | 9 (6-14) | <0.001 |
| Age group | *0 – 5 yrs.* | 358 | 8 (5-12) | 394 | 7 (5-11) | 0.040 |
|  | *6 – 18 yrs.* | 336 | 11 (7-15) | 534 | 10 (7-14) | 0.34 |
|  | *19 – 59 yrs.* | 360 | 10 (6-14) | 392 | 8 (5-14) | 0.013 |
|  | *60+ yrs.* | 54 | 10 (5-14) | 62 | 8 (5-13) | 0.75 |
| Gender | *Male* | 608 | 10 (6-15) | 682 | 9 (6-14) | 0.036 |
|  | *Female* | 394 | 10 (7-14) | 560 | 9 (5-14) | 0.43 |
| HH size | *<4 pp.* | 290 | 9 (6-14) | 332 | 8 (5-14) | 0.62 |
|  | *4 pp.* | 280 | 9 (6-14) | 314 | 8 (6-12) | 0.12 |
|  | *5 pp.* | 224 | 10 (6-14) | 334 | 8 (5-13) | 0.085 |
|  | *6+ pp.* | 310 | 11 (8-15) | 400 | 10 (6-14) | 0.37 |
| HH type | *Extended* | 498 | 10 (7-15) | 794 | 8 (6-14) | <0.001 |
|  | *Nuclear* | 606 | 10 (6-14) | 588 | 9 (6-14) | 0.87 |
| HH SES index | *Low* | 264 | 10 (6-15) | 528 | 8 (6-14) | 0.096 |
|  | *Medium* | 242 | 11 (7-15) | 580 | 9 (6-14) | <0.001 |
|  | *High* | 602 | 9 (6-14) | 274 | 9 (6-14) | 0.72 |
| Students | *Active* | 101 | 12 (9-16) | 333 | 11 (7-16) | 0.99 |
|  | *Non-active* | 991 | 10 (6-14) | 1045 | 8 (5-13) | <0.001 |
| Workers | *Active* | 76 | 10 (7-14) | 25 | 13 (7-15) | 0.61 |
|  | *Non-active* | 1016 | 10 (6-14) | 1353 | 9 (6-14) | 0.011 |
| Day type | *Weekday* | 340 | 10 (6-15) | 961 | 9 (6-14) | 0.34 |
|  | *School holiday* | 494 | 9 (6-14) | 5 | 3 (1-10) | 0.11 |
|  | *Weekend* | 274 | 10 (7-14) | 412 | 8 (6-12) | 0.0022 |
| Use of shadow | *Yes* | 564 | 9 (6-14) | 712 | 8 (6-12) | 0.72 |
|  | *No* | 544 | 10 (7-15) | 668 | 9 (5-14) | 0.0027 |
| Type of contact | *Physical* | 1001 | 6 (3-9) | 1321 | 6 (3-9) | 0.82 |
|  | *Non-physical* | 750 | 2 (0-5) | 901 | 2 (0-4) | 0.002 |

**Table B GEE model for the number of social contacts.** Coefficients (with respective semi-robust standard error and significance at 5%) for the association between the number of social contacts (overall and by setting of contact), according to GEEs with negative binomial distribution, Manicaland (Zimbabwe), 2013. Differently from Table 2 in the main text, we explore here the seasonal change in climatic conditions using the average temperature in the province.

| **Variable** | **Category** | **Overall** | **Home** | **School**^a^ | **Work**^a,b^ | **General**  **Comm.** |
| --- | --- | --- | --- | --- | --- | --- |
| **Intercept** |  | 1.99  (0.19)* | 1.66  (0.22)* | 1.14  (0.69) | 1.88  (1.39) | 1.32  (0.33)* |
| **Total time use (hours)** | *School* | 0.052  (0.0060)* | -0.036  (0.0067)* | 0.026  (0.025) | - | -0.0071  (0.015) |
| *Ref.: Home* | *Workplace* | 0.040  (0.0099)* | -0.031  (0.012)* | - | 0.14  (0.038)* | -0.026  (0.025) |
|  | *General*  *community* | 0.051  (0.0047)* | -0.023  (0.0048)* | -0.040  (0.029) | 0.096  (0.072) | 0.090  (0.0089)* |
| **Site of residence** | *Urban* | 0.11  (0.059) | 0.14  (0.064)* | -0.10  (0.15) | -0.53  (0.45) | 0.31  (0.11)* |
| **Gender** | *Female* | -0.13  (0.036)* | -0.18  (0.041)* | -0.022  (0.11) | -0.30  (0.31) | -0.049  (0.069) |
| **Age group** | *0 – 5 yrs.* | -0.23  (0.045)* | -0.13  (0.048)* | -0.26  (0.21) | - | -0.31  (0.088)* |
| *Ref.: 6 – 18 yrs.* | *19 – 59 yrs.* | -0.029  (0.048) | -0.056  (0.052) | -0.56  (0.38) | - | 0.17  (0.081)* |
|  | *60+ yrs.* | -0.025  (0.10) | 0.018  (0.13) | n.a. | - | 0.20  (0.18) |
| **Household size** |  | 0.035  (0.0093)* | 0.078  (0.010)* | 0.021  (0.035) | -0.20  (0.062)* | -0.023  (0.016) |
| **Socio-economic status**^c^ | *Medium* | -0.087  (0.048) | -0.12  (0.054)* | -0.33  (0.14)* | 0.63  (0.45) | -0.055  (0.080) |
| *Ref.: Low* | *High* | -0.081  (0.053) | -0.13  (0.062)* | -0.12  (0.14) | 0.46  (0.31) | -0.12  (0.099) |
| **Class size** | *Size* | **-** | - | 0.051  (0.021)* | - | **-** |
|  | *Size squared* | **-** | - | -0.0005  (0.00024)* | - | **-** |
| **Workplace size** | *Size* | - | - | - | 0.0098  (0.013) | - |
|  | *Size squared* | - | - | - | -0.000068  (0.00008) | - |
| **Day type** | *School holiday* | 0.011  (0.053) | -0.058  (0.059) | 0.019  (0.25) | 0.44  (0.34) | 0.15  (0.10) |
| *Ref.: Weekday* | *Weekend* | -0.0043  (0.039) | 0.020  (0.041) | -0.15  (0.20) | 0.46  (0.24) | 0.055  (0.073) |
| **Average monthly temperature** |  | 0.0041  (0.0013) | -0.00079  (0.015) | -0.035  (0.031) | -0.058  (0.088) | -0.019  (0.023) |
| **Sample size** |  | 2218 | 2207 | 380 | 62 | 1493 |

*: *p*<0.05.

^a^ For school and workplace, the analysis was performed on the subsamples of individuals who reported time use data for such settings.

^b^ *Total time use (school)*, and *Age* have been excluded from the model for the sake of convergence.

^c^ The *Socio-economic status* has been computed using data on household characteristics and owned assets [39].

**Fig A.** **Distribution of physical and non-physical contacts**. Number of physical (top row) and non-physical (bottom row) contacts in the study population in Manicaland, Zimbabwe (2013). a, e) Distribution of the overall number of contacts reported by participants. b, f) Boxplot (2.5%, 25%, 50%, 75%, and 97.5% quantiles) of the number of contacts stratified by age group of participant and by active participation to school or work: preschool (0–5 yrs.), school and no school (6–18 yrs.), work and no work (19–59 yrs.), and old age (60+ yrs.). c, g) Average number of contacts stratified by age group of participant and activity class and by setting of contact for the peri-urban township. d, h) As in c, g), but for the subsistence farming area.


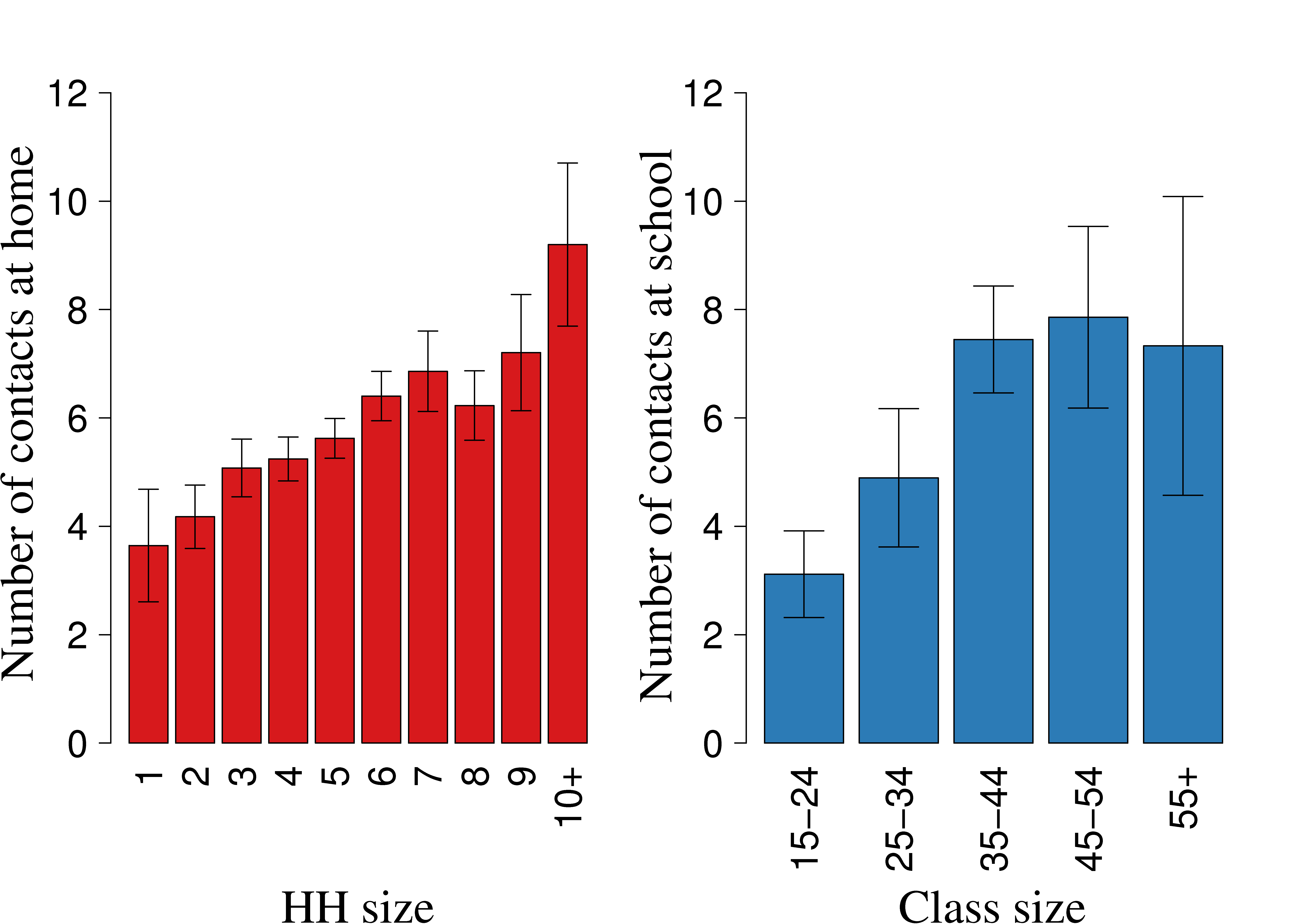


Fig B. Number of contacts by setting size. Average number of contacts at home by household size and at school by class size. Bars represent 95% CI around the average.

**Further stratifications of the social contact matrix (smoothed version)**

Fig C. Contact matrix with average number of contacts, by setting. Social contact matrices reporting the average number of contacts of participants in the *i*th age group with individuals in the *j*th age group, in different settings: (top left panel) at home, (top right panel) at school, (bottom left panel) at work, and (bottom right panel) in the general community. School holidays are excluded.

Fig D. Contact matrix with average number of contacts, by site. Social contact matrices reporting the average number of contacts of participants in the *i*th age group with individuals in the *j*th age group, by site of residence: (left panel) peri-urban township, (right panel) subsistence farming area. School holidays are excluded.

Fig E. Contact matrix with average number of contacts, by gender. Social contact matrices reporting the average number of contacts of participants in the *i*th age group with individuals in the *j*th age group by (left panel) females and (right panel) males. School holidays are excluded.

Fig F. Contact matrix with average number of contacts, by type of day. Social contact matrices reporting the average number of contacts of participants in the *i*th age group with individuals in the *j*th age group, (left panel) on working days, and (right panel) on weekends and during school holidays.

Fig G. Contact matrix with average number of contacts, by proximity of contact. Social contact matrices reporting the average number of contacts of participants in the *i*th age group with individuals in the *j*th age group: (a) physical contacts, and (b) non-physical contacts (conversational contacts with no skin-to-skin contacts). School holidays are excluded.

Fig H. Contact matrix with average number of contacts, by type of household. Social contact matrices reporting the average number of contacts of participants in the *i*th age group with individuals in the *j*th age group, in the household: (left panel) participants living in nuclear families (single, couples without children, parents with children), and (right panel) participants living in extended families. School holidays are excluded.

Fig I. Contact matrix with average number of contacts, observed vs. proportionate mixing. Social contact matrices reporting the average number of contacts of participants in the *i*th age group with individuals in the *j*th age group with (left panel) observed average number of social contacts in the two study sites (school holidays included), and (right panel) simulated number of social contact assuming proportionate mixing (school holidays included).

**Social contact matrices with observed number of contacts**

For the sake of comparison with the smoothed social contact matrices shown in the main manuscript (Fig 3 in the main manuscript), we report here the observed contact matrices with the same stratification (no bivariate smoothing applied). We can notice that, despite the numbers are a little different, the shape of the two types of matrix (smoothed and observed) are very similar to each other.

Fig J. Observed contact matrix with average number of contacts, by country. Social contact matrices, obtained from observed contacts, reporting the average number of contacts (no school holidays) of participants in the *i*th age group with individuals in the *j*th age group: (left panel) overall social contacts in the two study sites, and (right panel) overall social contacts in Italy (data from the Polymod study [5]).

**Social contact matrices with contact rates**

For the sake of comparison with the smoothed social contact matrices shown in the main manuscript (Fig 3 in the main manuscript), we display here the contact matrices with the same stratification, but reporting the contact rates rather than the average number of contacts. Contact rate matrices, unlike the latter ones, are perfectly symmetrical, as they report the rate at which a person aged *i* encounters a person aged *j*, and the values of contact rates are dependent on the reference population, i.e., the larger the population, the smaller the contact rates. This explains why the contacts rates in the study population in Manicaland (left panel) are larger than those in Italy (right panel). The two matrices appear quite different from each other. In Manicaland, age assortativeness is particularly intense among working-age adults (30-49 years) and among the elderly (more than 60 years). On the contrary, in Italy the assortativeness by age is more evident among school age children and reduce drastically as age increases. Moreover, in Manicaland we see a certain degree of contact between the elderly and the young people (10-19 years), while in Italy this intergenerational pattern is more evident between adults aged 40-49 years (possibly parents) and young people aged 10-19 years (possibly children).

Fig K. Contact matrix with contact rates, by country. Social contact matrices, obtained from observed contacts, reporting the rate at which a participant in the *i*th age group encounters with an individual in the *j*th age group: (left panel) social contact rates in the two study sites, and (right panel) social contact rates in Italy (data from the Polymod study [5]).

**References**

1. Liang K-Y, Zeger SL. Longitudinal Data Analysis Using Generalized Linear Models. Biometrika. 1986;73: 13–22.

2. Hens N, Goeyvaerts N, Aerts M, Shkedy Z, Van Damme P, Beutels P. Mining social mixing patterns for infectious disease models based on a two-day population survey in Belgium. BMC Infect Dis. 2009;9: 5. doi:10.1186/1471-2334-9-5

3. Vyas S, Kumaranayake L. Constructing socio-economic status indices: how to use principal components analysis. Health Pol Plan. 2006;21: 459–468. doi:10.1093/heapol/czl029

4. Gregson S, Garnett GP, Nyamukapa CA, Hallett TB, Lewis JJC, Mason PR, et al. HIV Decline Associated with Behavior Change in Eastern Zimbabwe. Science. 2006;311: 664–666. doi:10.1126/science.1121054

5. Mossong JR, Hens N, Jit M, Beutels P, Auranen K, Mikolajczyk RT, et al. Social contacts and mixing patterns relevant to the spread of infectious diseases. Riley S, editor. PLoS Med. 2008;5: 0381–0391. doi:10.1371/journal.pmed.0050074
