# Supplementary material for: Social Contact Structures and Time Use Patterns in the Manicaland Province of Zimbabwe
Source: PLoS One. 2017 Jan 18;12(1):e0170459. doi: 10.1371/journal.pone.0170459 (PMC5242544; doi:10.1371/journal.pone.0170459)
Supplement: S4 Text — Further stratifications for the time use patterns. (DOCX) [file pone.0170459.s007.docx]

**S4 Text**

**Analysis of Time Use Data**

The following plots are additional stratifications of the Time Use data, for both Manicaland study population and Italy [1], for comparative purposes. In particular, we present daily routines for the four considered age groups, stratifying the data by the day of the week in which the diary was kept (i.e., weekdays vs. weekends/school holidays).





Fig A. Time use pattern on weekdays and during weekends/school holidays, in Manicaland (Zimbabwe), 2013. The plots show the proportion of individuals present in different settings (home, school, workplace, general community) at different hours of the day during weekdays (Monday to Friday, 5:00 am - 10:00 pm) (top row) and weekends/school holidays (5:00 am - 10:00 pm) (bottom row) by age group of participant, in the study population in Manicaland, Zimbabwe (2013)





Fig B. Time use pattern on weekdays and during weekends/school holidays, in Italy, 2003. The plots show the proportion of individuals present in different settings (home, school, workplace, general community) at different hours of the day during weekdays (5:00 am - 10:00 pm, Monday to Friday, from October to May) and weekends/school holidays (5:00 am - 10:00 pm, weekends all year, and school holidays, from June to September) by age group of participant, in Italy (2003) [1].

**References**

1. Romano MC, editor. Time Use in Daily Life. Istat; 2008 May pp. 1–333. Report No.: 35.
